# Photogeneration of Hydrogen in Water via Self‐Assembly‐Induced Colloidal Covalent Organic Framework Particles

**DOI:** 10.1002/smsc.202500450

**Published:** 2026-03-06

**Authors:** Axelle Larrieu, Sabuj Kanti Das, Aurelien Viterisi, Laurent Billon

**Affiliations:** ^1^ Bio‐inspired Materials Group: Functionalities & Self‐assembly Institut des Sciences Analytiques et Physico‐chimie pour l'environnement et les matériaux Universite de Pau et des Pays de l’Adour 64000 Pau France

**Keywords:** colloids, covalent organic frameworks, hydrogen evolution reaction, photocatalysis, surfactant‐free process

## Abstract

The synthesis of colloidal imine‐linked Covalent Organic Framework nanoparticles using a polymeric growth‐blocking agent that facilitates both *in situ* colloidal stabilization and size control. This new surfactant‐free method, called Self‐Assembly‐Induced Colloidal Covalent Organic Frameworks (SAI2COF), is inspired by Polymerization‐Induced Self‐Assembly (PISA), largely used to generate colloidal emulsions. The optimized formulation, *I‐COF‐Poly‐0.5*, shows excellent colloidal stability in aqueous media under neutral and acidic conditions, along with enhanced light absorption and π‐conjugation. Photocatalytic hydrogen evolution demonstrates that *I‐COF‐Poly‐0.5* is an efficient light‐driven catalyst for at least 24 h, with performance strongly influenced by the sacrificial electron donor (SED), photocatalyst concentration, and metal cocatalyst. Sodium ascorbate is identified as the most effective SED for sustained H_2_ production, whereas triethanolamine induces rapid deactivation. An optimal COF concentration of 0.1 g L^−1^ provides the best balance between light penetration and catalytic efficiency. Among tested cocatalysts, platinum salts (H_2_PtCl_6_, K_2_PtCl_6_) significantly outperform AgNO_3_, which forms less active silver nanoparticles. Single‐particle induced coupled plasma mass spectrometry confirms the formation of hybrid colloidal COF particles containing in situ photo‐generated metal nanoparticles. Overall, this surfactant‐free approach affords stable, dispersed COF photocatalysts for clean hydrogen production over 24 h without aggregation and paves the way for further optimization.

## Graphical abstract

A surfactant‐free strategy using a functional Poly(DiMethylAminoEthylMethAcrylate) polybase enables stable colloidal imine‐linked COFs formation via self‐assembly‐induced process. A dual effect is demonstrated with the control in size and stabilization in colloidal state of the COFs in situ synthesized. The optimized *I‐COF‐Poly‐0.5* formulation shows excellent colloidal stability in water, strong light absorption, and linear hydrogen production over 24h without any aggregation.

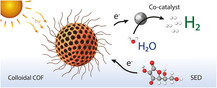

## 1 Introduction

In the current context of energy transition, the production of green hydrogen (H_2_) through photocatalysis represents a promising pathway to addressing environmental challenges.^[^ ^1^, ^2^, ^3^, ^4^ ^]^ It enables solar energy to be converted directly into a clean energy carrier, without CO_2_ emissions. Given that the solar energy received at the Earth's surface far exceeds global energy needs, this sustainable approach opens up major opportunities for reducing dependence on fossil fuels and contributing to the decarbonization of the energy and industrial sectors.

Among the various photocatalytic systems,^[^ ^3^, ^5^, ^6^, ^7^, ^8^, ^9^, ^10^ ^]^ covalent organic frameworks (COFs) have garnered increasing interest in recent years.^[^ ^3^, ^11^, ^12^, ^13^, ^14^, ^15^ ^]^ These materials are particularly attractive because their structural and functional properties can be finely tuned by selecting appropriate precursors for their synthesis. Key characteristics such as pore size, surface functionalities, electronic affinity, and light absorption properties can be precisely adjusted through the choice of building blocks.^[^ ^14^ ^]^ Imine‐linked COFs, in particular, are chemically and thermally stable, with good resistance in aqueous environments, making them promising candidates for photocatalytic applications in water.^[^ ^14^, ^16^, ^17^, ^18^, ^19^ ^]^ However, their rapid crystal growth significantly reduces their solubility/dispersion in organic media, leading to rapid precipitation in solution and limiting control of their morphology and processability.^[^ ^20^, ^21^ ^]^ A promising strategy to overcome this limitation is the synthesis of colloidal COF dispersions.^[^ ^22^, ^23^, ^24^, ^25^, ^26^, ^27^, ^28^, ^29^ ^]^ To this end, the use of a growth‐blocking agent presents an effective approach to controlling COF morphology. By restricting crystal growth along specific spatial directions, this agent stops the COF growth and then can stabilize its core surrounded by the blocking agent. For optimal efficiency, the blocking agent should exhibit reactivity comparable to one of the COF precursors while remaining monofunctional to terminate the crystallization process.^[^ ^27^, ^30^, ^31^ ^]^ This approach has already been applied to boronic ester COFs. Salaün et al. developed a method for synthesizing colloidal COF‐polymer hybrids by incorporating catechol‐functionalized polymers during COF‐5 synthesis.^[^ ^30^ ^]^ The end‐functionalized polymer acts as a modulator, competing with the HHTP (2,3,6,7,10,11‐hexahydroxytriphenylene) building block for reaction with its complementary counterpart. Their study demonstrated that catechol‐poly(n‐butyl acrylate) leads to quasispherical COF crystallites, while catechol‐poly(N‐IsoPropylacrylAMide) results in raspberry‐like COF‐polymer particles. Recently, our team explored the stabilization of imine‐linked COFs in colloidal form by introducing a polymeric growth‐blocking agent.^[^ ^31^ ^]^ Specifically, an amine end‐functionalized Poly(2‐(DiMethylAmino)Ethyl MethAcrylate) (PDMAEMA) was selected as a growth inhibitor for imine COFs. This hydrophilic polymer enables the stabilization of colloidal imine COFs in aqueous media, facilitating their application in proton reduction for hydrogen production. In addition, as a pH‐sensitive polymer, PDMAEMA could further enhance hydrogen generation by improving proton transport to COF active sites.^[^ ^32^, ^33^, ^34^ ^]^

The use of colloidally stabilized organic nanoparticles (NPs) has already been explored for hydrogen production.^[^ ^29^, ^35^, ^36^ ^]^ Indeed, Wang et al. demonstrated that poly[(9,9′‐dioctylfluorenyl‐2,7‐diyl)‐*co*‐(1,4‐benzo‐{2,1′,3} thiadiazole)] (PFBT) nanoparticles, stabilized by an amphiphilic copolymer (polystyrene‐*comb*‐poly(ethylene oxide)), could serve as efficient photocatalysts for hydrogen evolution.^[^ ^37^ ^]^ Their study revealed that maintaining NPs in colloidal form significantly enhanced their photocatalytic performance. Similarly, Cloutet and coworkers showed that stabilizing donor–acceptor–donor trimers with amphiphilic block copolymers led to a substantial increase in photocatalytic activity compared to trimers dispersed in solution without stabilization.^[^ ^38^ ^]^

**Scheme 1:**
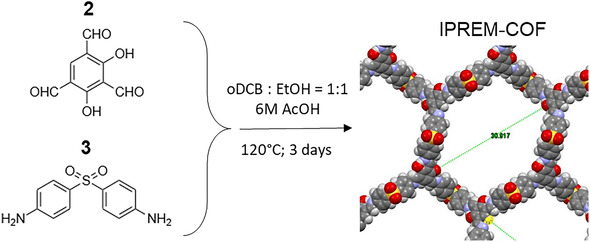
Synthetic scheme of IPREM‐COF also called *I‐COF*.

In this work, we introduce a new COF material synthesized from 2,4‐dihydroxybenzene‐1,3,5‐tricarbaldehyde and 4,4′‐diaminobiphenyl sulfone, designated as IPREM‐COF (later abbreviated to *I‐COF*) (**Scheme** **1**).

This imine‐based COF offers several advantages. First, it contains hydroxyl groups located close to the imine bonds (C=N), enabling the formation of intramolecular hydrogen bonds (O—H····N=C) within the network. These interactions enhance both the chemical stability and crystallinity of the COF.^[^ ^39^ ^]^ By reinforcing the planarity of the structure, they also promote stronger interlayer interactions during stacking.^[^ ^40^ ^]^ Additionally, IPREM‐COF incorporates sulfone groups, which introduce heteroatoms into the framework. These heteroatoms create partial charge separations, leading to polarized bonds that facilitate the destabilization of stable molecules such as water, thereby enhancing its oxidation.^[^ ^41^ ^]^

Furthermore, the presence of sulfone groups extends the COF's light absorption range (λ ≤ 600 nm), improving its ability to absorb visible light for photocatalysis. The sulfone functionality also improves pore wettability, facilitating interactions between COF and water, and thus enhancing photocatalytic water reduction.^[^ ^16^, ^42^ ^]^

We propose the synthesis of a colloidal COF particle IPREM‐COF *via* the addition of a macromolecular growth‐blocking agent, also recently described by our group as a surfactant‐free concept and so‐called self‐assembly‐induced colloidal COF (SAI2COF).^[^ ^43^ ^]^ This new concept involves in‐situ stabilization of COF nanoparticles with a water‐soluble macromolecular functional blocking agent inspired from the polymerization‐induced self‐assembly (PISA)^[^ ^37^ ^]^ largely used for the generation of colloidal polymeric emulsion. This synthetic method prevents the rapid precipitation of COF in solution, ensuring greater stability in aqueous suspension and facilitating its use in photocatalysis. The resulting colloidal COF particles were then tested in presence of different type of the sacrificial electron donor (SED), metal cocatalysts, for hydrogen production (H_2_).

## 2 Experimental Section

### 2.1 Materials

4,4′‐diaminobiphenyl sulfone (98%) and trifluoroacetic acid (99%) were supplied by ABCR. 1,2‐dichlorobenzene was from Alfa Aesar. Glacial acetic acid (≥99%), tetrahydrofuran (THF), ≥99%, stabilized with BHT), 1,4‐dioxane (≥99%, stabilized), absolute ethanol (≥99.8%), dimethylformamide (DMF), and acetone were from VWR. Sodium L‐ascorbate (≥99.0%), triethanolamine (TEOA), silver nitrate (AgNO_3_), potassium hexachloroplatinate(IV) (98%), and resorcinol (98%) were purchased from Sigma‐Aldrich. L‐ascorbic acid (≥99.5%) was from Apollo Scientific. Hydrogen hexachloroplatinate(IV) hexahydrate (H_2_PtCl_6_, 6 H_2_O) was supplied by TCI. Hexamethylenetetramine (99%) was purchased from Acros Organics. Hydrochloric acid (37% fuming) was from Supelco. The ultrapure water (18 mΩ.cm^−1^ or 55 μS.cm^−1^) used was filtered by a Millipore water purification system (Merck, Darmstadt, Germany). pH buffer solution pH 7 and pH 3 were purchased from Carl Roth.

Amine end‐functionalized Poly(DiMethylAmino)Ethyl MethAcrylate was synthesized by previously reported procedure.^[^ ^31^ ^]^

### 2.2 Synthesis

#### 2.2.1 Synthesis of Aldehyde Precursor: 2,4‐Dihydroxybenzene‐1,3,5‐Tricarbaldehyde

Resorcinol (3.6 g, 32.7 mmol) and hexamethylenetetramine (10 g, 71.3 mmol) were dissolved in 35 mL of trifluoroacetic acid. The solution was degassed under nitrogen and stirred at 130 °C for 16 h, then heated to 150 °C for 3 h. After cooling to 120 °C, 55 mL of an aqueous HCl solution (3 mol.L^−1^) was added, and the mixture was heated to 105 °C for a further 30 min. The resulting yellow powder was then filtered, washed with water and ethanol, and dried under vacuum. Finally, the powder was purified by recrystallization in hot DMF.

#### 2.2.2 Synthesis of IPREM‐COF by Solvothermal Route: I‐COF

2,4‐dihydroxybenzene‐1,3,5‐tricarbaldehyde (38.8 mg, 0.2 mmol) and 4,4′‐diaminobiphenyl sulfone (74.5 mg, 0.3 mmol) were introduced into a Schlenk tube in 3 mL of a 1:1 oDCB:EtOH solvent mixture. Then, 0.2 mL of an aqueous acetic acid solution (6 mol.L^−1^) was added. After three freeze‐pump‐thaw degassing cycles, the mixture was heated to 120 °C for 3 days under static conditions. The resulting orange powder (*I‐COF*) was filtered, washed with water, ethanol, THF, and acetone, and dried under vacuum.

#### 2.2.3 Synthesis of IPREM‐COF Under Mild Conditions Without Blocking Agent: I‐COF‐80 °C

2,4‐dihydroxybenzene‐1,3,5‐tricarbaldehyde (27.3 mg, 0.14 mmol) and 4,4′‐diaminobiphenyl sulfone (52.5 mg, 0.21 mmol) were dissolved in 15 mL of 1,4‐dioxane. The mixture was degassed under nitrogen and stirred at 80 °C for 8 h. The resulting powder was filtered, washed with water, ethanol, THF, and acetone, and dried under vacuum.

#### 2.2.4 Synthesis of Colloidal IPREM‐COF with a Macromolecular Growth‐Blocking Agent by SAI2COF

**Scheme 2:**
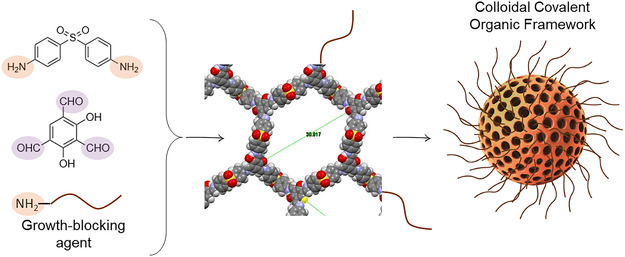
Schematic diagram of the synthetic strategy for a colloidal COF using a macromolecular growth‐blocking agent.

A mixture of 2,4‐dihydroxybenzene‐1,3,5‐tricarbaldehyde (27.3 mg, 0.14 mmol), 4,4′‐diaminobiphenyl sulfone (52.5 mg, 0.21 mmol), and functionalized PDMAEMA (molecular weight: 6 000 g.mol^−1^, *D* = 1.35) was dissolved in 15 mL of 1,4‐dioxane (**Scheme** **2**).^[^ ^44^ ^]^ Three different ratios of macromolecular growth‐blocking agent, amine end‐functionalized PDMAEMA, were tested and then introduced. The different molar ratios of PDMAEMA to the aldehyde precursor tested correspond to 0.01, 0.1, and 0.5 molar equivalents per 1 mole of aldehyde precursor, corresponding to COFs named *I‐COF‐Poly‐0.01*, *I‐COF‐Poly‐0.1*, and *I‐COF‐Poly‐0.5*, respectively (see **Table** **1**). The mixture was then degassed under nitrogen and stirred at 80 °C for 8 h.

**Table 1:** Experimental conditions to control COF growth with an amine end‐functionalized PDMAEMA.

	Number of moles [mmol]		
Sample name	Aldehyde precursor	Amine precursor	Polymer
*I‐COF‐Poly‐0.01*	0.14	0.21	0.0014
*I‐COF‐Poly‐0.1*	0.14	0.21	0.014
*I‐COF‐Poly‐0.5*	0.14	0.21	0.070

#### 2.2.5 Solubility and Redispersion Tests of Colloidal COFs in Water

After the reaction, the COF solution was left to dry at room temperature to allow dioxane evaporation. Once dried, 10 mg of the resulting COF powder was redispersed in 5 mL of an aqueous solution buffered at pH = 3 and another buffered at pH = 7 (Figure S1, Supporting Information).

### 2.3 Methods

#### 2.3.1 Infrared Spectroscopy

Infrared spectra were obtained using a Thermo Fisher Nicolet IS50 spectrophotometer in attenuated total reflectance (ATR) mode. Each sample was scanned 64 times over the 4000 and 400 cm^−1^ range, at room temperature. Data were collected using OMNIC 9.8 software.

#### 2.3.2 UV–Visible Spectroscopy

UV–visible spectra were measured using a Shimadzu UV‐2450 spectrophotometer. Measurements were taken using two cuvettes with optical path lengths of 1 cm and 0.2 cm (for photocatalysis solutions). Spectra were collected between 200 and 800 nm with a 0.5 nm step. For solid‐state UV measurements, the powders were analyzed using a UV–visible spectrophotometer (Perkin Elmer 860 with dual beam) over the 250–800 nm with a 1 nm step and a 100 nm min^−1^ scan rate. Measurements were performed using a 15 cm‐diameter integrating sphere coated with a 4 mm layer of Spectralon. The sample holder was placed in a horizontal position.

#### 2.3.3 Powder X‐Ray Diffraction (PXRD)

PXRD measurements were carried out using a Rigaku FRX diffractometer equipped with a 3 kW rotating copper anode (Kα radiation) and a 3‐circle partial chi goniometer (Rigaku). Samples were analyzed over a 2*θ* range of 0°–50°. The *I‐COF* sample was analyzed separately using a Bruker D8 Advance diffractometer with copper Kα radiation, also within a 2*θ* range of 0°– 50°.

#### 2.3.4 Zeta Potential and Dynamic Light Scattering (DLS)

DLS measurements were carried out on a Nano‐ZS Zetasizer ZEN3600 (Malvern Instruments) at a 173° detection angle. The instrument was equipped with a 4.0 mW He‐Ne laser (633 nm wavelength). Solutions were prepared at a concentration of 0.5 g.L^−1^. Measurements were conducted at room temperature. Hydrodynamic diameters were analyzed based on number distributions. Zeta potential analysis was carried out using an MPT‐2 autotitrator system assessing the stability of the dispersion across a pH range of 2–12. pH adjustments were made using a 1 mol.L^−1^ NaOH and 1 mol.L^−1^ HCl buffer solutions.

#### 2.3.5 Scanning Electron Microscopy (SEM)

SEM images were acquired using a Hirox SH‐3000 microscope with an accelerating voltage of 25 kV and a secondary electron detector. Powder samples were fixed onto horizontal sample holders using double‐sided carbon adhesive and coated with a 10 nm gold layer using a Desk V metallizer (30 mA, 60 s). SEM images presented in Figure 7 were obtained using a ThermoFisherScientific Apreo 2S microscope with an accelerating voltage of 2 kV. Solutions were dropcasted onto silicon wafers and dried at room temperature. All samples were coated with a thin 4 nm layer of platinum using a Cressington 208 HR metallizer. Scanning transmission electron microscopy (STEM) images were taken using a ThermoFisherScientific Apreo 2S scanning electron microscope equipped with a STEM‐in‐SEM detector, with an accelerating voltage of 30 kV and a current of 0.1 nA. A 1.5 μL drop of the solution containing the dispersed sample was deposited on a Cu grid (200 mesh, formvar/C, type B, from Eloise). After 1 min, the excess drop was removed using absorbent paper.

#### 2.3.6 Single Particle‐Induced Coupled Plasma Mass Spectrometry (SP‐ICP‐MS)

SP‐ICP‐MS analyses were performed using a PerkinElmer NexION 5000 instrument equipped with a single nanoparticle application module (PerkinElmer, Shelton, CT) for nanoparticle detection and quantification. The instrument and acquisition parameters are summarized in Table S1, Supporting Information. Operating at low nanoparticle concentrations and high‐frequency data acquisition, the SP‐ICP‐MS can provide information on the number concentration of a suspension containing nanoparticles as well as their size distribution in a single measurement.^[^ ^45^ ^]^ Samples were diluted 100 000 times with ultrapure water and introduced directly into the SP‐ICP‐MS.

#### 2.3.7 N_2_ Sorption Measurements

Were performed using an Autosorb iQ at 77 K from Quantachrome. The samples were activated at 120 °C during 20 h.

#### 2.3.8 Photocatalytic Hydrogen Evolution

*Preparation of colloidal dispersions.* Colloidal COF particles were dried and redispersed in 10 mL ultrapure water. An electron‐donating sacrificial agent was added at a concentration of 0.1 mol.L^−1^, then 5 μL of an aqueous solution of an 8 wt% cocatalyst was added. *Photocatalysis setup.* All photocatalysis experiments were carried out in a glass cell with a quartz window. This cell was irradiated at 1 SUN from below, through the quartz window, with a light‐emitting diode (LED) lamp (10 W, LCFOCUS, China). The emission spectrum of the lamp is shown in Figure S2, Supporting Information. The distance between the lamp and the cell was calibrated to 1 SUN using a reference photodiode. The reference photodiode was placed at the quartz window, and the height of the cell was adjusted to obtain a measurement of the photodiode short‐circuit current equal to the calibration value. The system was stirred using a vertical magnetic stirrer on the side of the cell. The temperature was kept constant at 25 °C using a double wall screwed to the bottom of the cell (Figure S3, Supporting Information). During each experiment, the cell was purged for 20 min under N_2_ at a flow rate of 35 mL min^−1^. The first sample was collected after 10 min of illumination and was used as a blank, followed by a sample every 3 h for 24 h. *Quantification of hydrogen production*. The hydrogen produced was identified and quantified by gas chromatography (GCMS‐QP2010‐plus, Shimadzu, Japan) equipped with a packed column (ShinCarbon ST, 2 m; 0.53 mm, mesh 80/100, Restek, USA). Helium (6.0, Alphagaz 2, AirLiquide) was used as the carrier gas. A dielectric barrier discharge ionization detector (BID, Shimadzu, DL < 0.1 ppm) was used to quantify the dihydrogen produced. Hydrogen production was monitored using an automated sampling system developed in the laboratory. In this system, solenoid valves are operated at regular intervals to transfer a sample from the photocatalysis cell headspace (1.2 mL) into a GC sampling loop before being injected into the GC column.

## 3 Results and Discussion

### 3.1 Self‐Assembly‐Induced Colloidal COF Particles

IPREM‐COF was initially synthesized *via* a solvothermal route (*I‐COF*) to serve as a reference material, ensuring optimal crystallization conditions. During this process, precipitation occurs immediately upon the addition of the aqueous acetic acid solution to the reaction mixture. To explore alternative synthesis conditions, IPREM‐COF was also synthesized under milder conditions, without an acid catalyst, at 80 °C for 8 h (*I‐COF‐80 °C*). These conditions were selected to match those used in the presence of the growth‐blocking agent, allowing *I‐COF‐80 °C* to serve as a control to assess the impact of the blocking agent on the final material's properties. Under these conditions, precipitation was observed within the first hour of the reaction. A macromolecular growth‐blocking agent, amino end‐functionalized PDMAEMA, was then introduced to elaborate by the SAI2COF process, colloidal COF particles. Three different molar ratios of PDMAEMA to the aldehyde precursor were tested: 0.01, 0.1, and 0.5 molar equivalents per 1 mole of aldehyde precursor, corresponding to COFs named *I‐COF‐Poly‐0.01*, *I‐COF‐Poly‐0.1*, and *I‐COF‐Poly‐0.5*, respectively. While *I‐COF‐Poly‐0.01* and *I‐COF‐Poly‐0.1* precipitated within the first hour, *I‐COF‐Poly‐0.5* remained stable throughout the 8‐hour reaction. Although sedimentation was observed after several weeks, the redispersion remained stable for several days upon stirring. Figure S4, Supporting Information, presents photographs of the reaction mixtures after 24 h. Despite precipitation in *I‐COF‐Poly‐0.01* and *I‐COF‐Poly‐0.1*, part of the COF appears to be stabilized by the macromolecular blocking agent, as indicated by the retention of the characteristic orange color in the soluble fraction, similar to *I‐COF*. These four syntheses were further monitored in the liquid phase *via* UV–visible spectroscopy. The obtained precipitates were characterized in terms of their composition, crystallinity, light absorption range, and morphology. In the case of *I‐COF‐Poly‐0.5*, where no precipitate was formed, the reaction mixture was dried, and the resulting powder was compared with other COFs.

#### 3.1.1 Light Absorption Range

**Figure 1:**
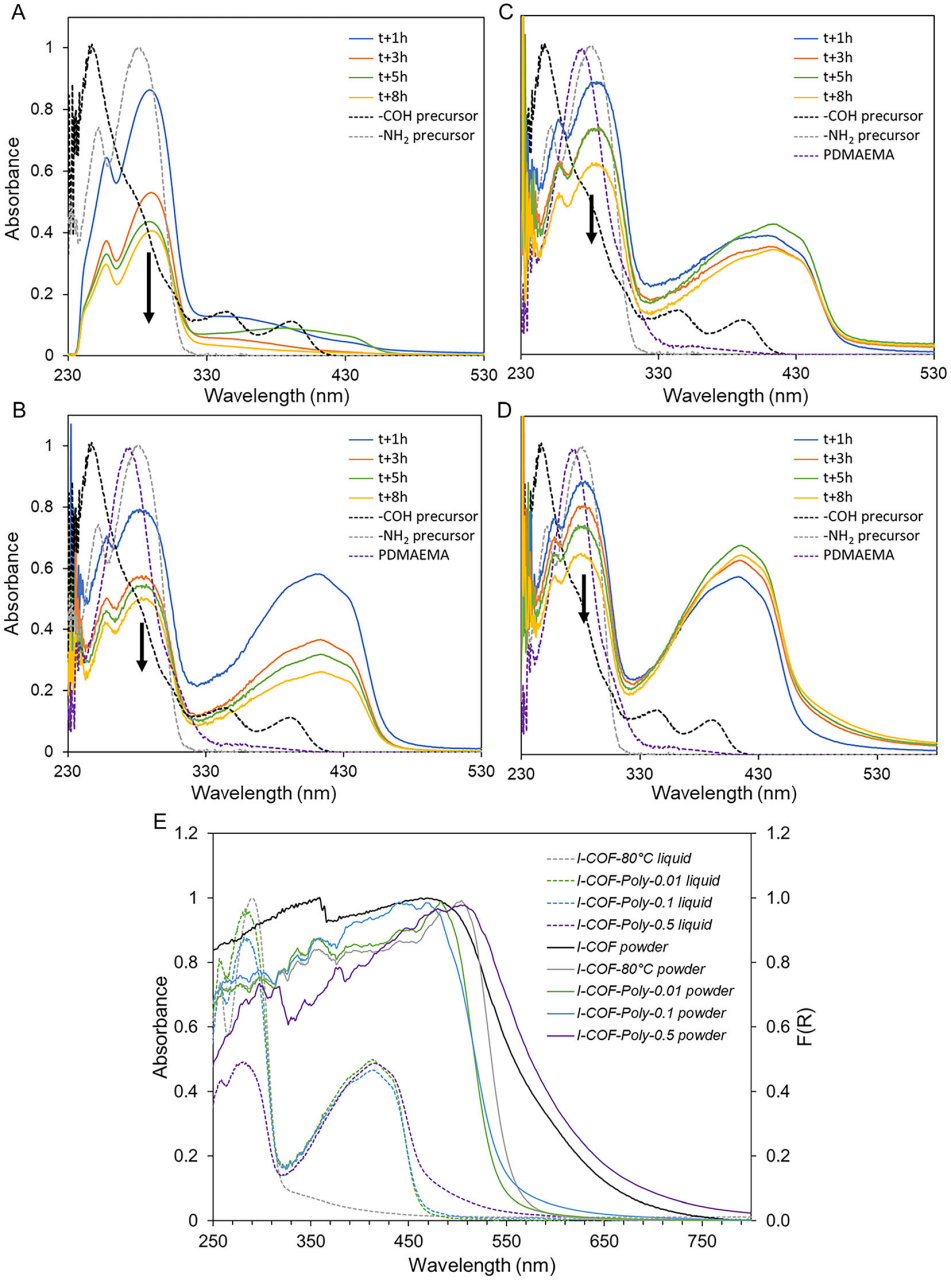
UV–visible spectra of the reaction mixture at different times for syntheses A) *I‐COF‐80 °C*, B) *I‐COF‐Poly‐0.01*, C) *I‐COF‐Poly‐0.1*, and D) *I‐COF‐Poly‐0.5*. E) Comparison of liquid‐phase UV–visible spectra at *t* + 8 h (dotted lines) and UV‐solid spectra of precipitates (solid lines) from *I‐COF* (black), *I‐COF‐80 °C* (gray), *I‐COF‐Poly‐0.01* (green), *I‐COF‐Poly‐0.1* (blue), and *I‐COF‐Poly‐0.5* (violet) syntheses.

**Figure** **1** presents the UV–visible spectra of the reaction mixture sampled at different times (after 1, 3, 5, and 8 h of reaction) for the different syntheses.

In the absence of a blocking agent (*I‐COF‐80 °C* synthesis, Figure 1A), the characteristic absorption peaks of the amine precursor (4,4′‐diaminobiphenyl sulfone) at 255 and 290 nm gradually decrease over time (Figure 1A), with a sharp drop in absorbance between 1 and 3 h, followed by a slight decrease between 3 and 8 h. This indicates that the amine precursor is being progressively consumed in the liquid phase as it participates in COF network formation. In addition, a new adsorption peak appears around 410 nm after 1 h of reaction, indicating the formation of conjugated motifs. However, the intensity of this absorption band remains relatively unchanged over time, suggesting that only a small proportion of COFs remain in solution. Instead, these newly formed conjugated structures rapidly reach a size too large for colloidal stability and precipitate as solid particles. This aligns with experimental observations, as a precipitate is already visible in the reaction mixture after just 1 h.

In the presence of a macromolecular blocking agent, the spectra (Figure 1B–D) similarly show a decrease in absorbance at 255 and 290 nm over time, confirming the consumption of the amine precursor. However, the absorption peak at 410 nm, characteristic of COF conjugation, is significantly more intense compared to the synthesis without a blocking agent. This suggests that the presence of a polymer enables better stabilization of COF units in solution.

Furthermore, by normalizing the spectra to the peak at 290 nm, differences in the evolution of the 410 nm absorption band become apparent depending on the polymer ratio introduced (Figure S5B–D, Supporting Information). Indeed, in the presence of a low polymer ratio (*I‐COF‐Poly‐0.01*), the peak decreases over time, indicating that the COFs do not remain stabilized and rapidly precipitate. For an intermediate polymer ratio (*I‐COF‐Poly‐0.1*), absorbance increases up to 5 h before slightly decreasing between 5 and 8 h, suggesting that this blocking agent concentration stabilizes only a limited number of COF units, beyond which precipitation occurs. Finally, for the highest polymer ratio (*I‐COF‐Poly‐0.5*), absorbance at 410 nm increases until the end of the 8 h reaction time, indicating sustained COF stabilization in solution.

Moreover, the absorbance ratio between the amine precursor peak (290 nm) and the COF peak (410 nm) confirms the progressive conversion of the precursor into the COF network (Figure S5D, Supporting Information). The *I‐COF‐Poly‐0.5* synthesis not only produces a higher amount of stabilized COF units in solution but also leads to more extended conjugation, observed by the redshift of the peak tail. Indeed, while COFs from *I‐COF‐Poly‐0.01* and *I‐COF‐Poly‐0.1* absorb up to 480 nm, those from *I‐COF‐Poly‐0.5* exhibit absorption up to 530 nm (Figure 1E). In the end, *I‐COF‐Poly‐0.5* seems to be the most favorable synthesis condition for stabilizing highly conjugated COF networks in solution, and thus for the elaboration of colloidal COF particles.

The light absorption properties of the precipitate materials were also compared (Figure 1E). While the solvothermal synthesis (*I‐COF*) resulted in a material capable of absorbing light up to 730 nm, *I‐COF‐80 °C*, *I‐COF‐Poly‐0.01*, and *I‐COF‐Poly‐0.1* syntheses led to COFs with absorption limited to 600 nm. On the other hand, *I‐COF‐Poly‐0.5* synthesis yields an absorption range similar to *I‐COF*. This difference can be attributed to variations in the extension of conjugation within the COF networks. Indeed, *in I‐COF‐80 °C*, *I‐COF‐Poly‐0.01*, and *I‐COF‐Poly‐0.1* *synthesis*, the COFs precipitate very rapidly, preventing the formation of large conjugated networks, also due to less favorable synthesis conditions than *I‐COF*. Conversely, for *I‐COF‐Poly‐0.5*, the initial reaction of the growth‐blocking agents with the COF building blocks favors their solubility and then their reactivity in solution, and stabilizes the reactive species for longer by preventing their precipitation. The colloidal stabilization enabled the formation of larger conjugated colloidal COF structures, resulting in a broader absorption range slightly higher than that of the reference COF. Indeed, a bandgap value of 1.97 eV (@ 630 nm) is obtained for *I‐COF‐Poly‐0.5* to be compared to a value of 2.03 eV (@610 nm) for *I‐COF* (Figure S6, Supporting Information). For the other compositions in polymer *I‐COF‐Poly‐0.01* and *I‐COF‐Poly‐0.1*, the bandgap value is estimated to 2.34 eV (@530 nm). So, the *I‐COF‐Poly‐0.5* would be interested to be used as a photocatalyst for H_2_ evolution if colloidally stable in water.

#### 3.1.2 Chemical Composition

**Figure 2:**
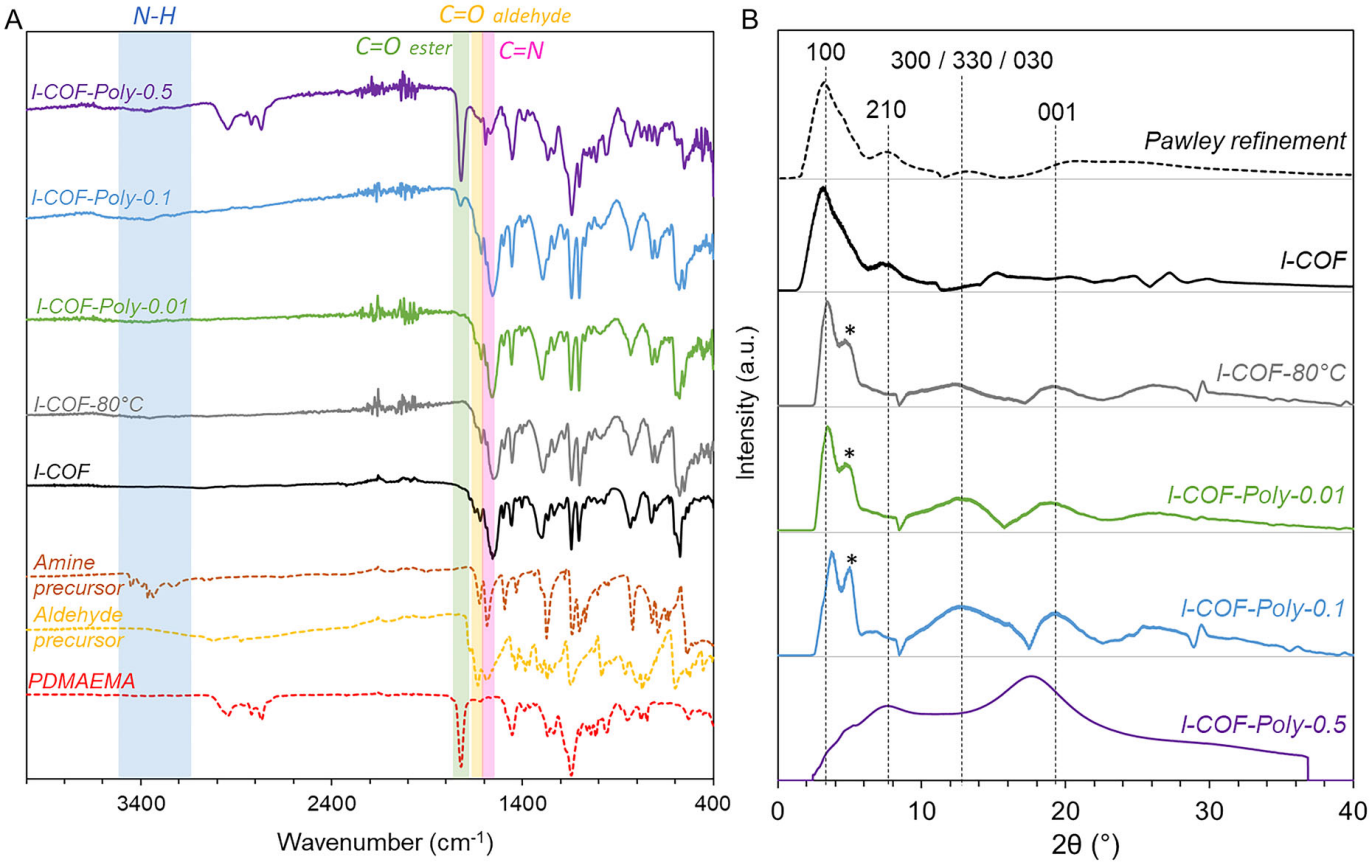
A) Infrared spectra of COFs and their precursors. B) Diffractograms of COFs obtained *via* the different syntheses and identification of crystal planes using a Pawley refinement.

The chemical composition of the different precipitates was compared by infrared spectroscopy (**Figure** **2**A).

Regardless of the synthesis conditions, the COF spectra show the disappearance of the N—H bond peaks (3210–3450 cm^−1^) from the amine precursor. Similarly, the absorption band corresponding to the C=O bond of the aldehyde precursor (1680 cm^−1^) has disappeared, except in the case of COF *I‐COF*, where a slight residual peak remains (see Figure S7 zoom, Supporting Information). In addition, a new absorption band appears at 1619, 1614, 1617, 1618, and 1622 cm^−1^ on the spectra of *I‐COF‐Poly‐0.5*, *I‐COF‐Poly‐0.1*, *I‐COF‐Poly‐0.01*, *I‐COF‐80 °C*, and *I‐COF*, respectively, confirming the formation of imine bonds (see Figure S7 zoom, Supporting Information). These results confirm that the precursors were fully consumed, regardless of the reaction conditions, and reacted with each other to form a COF network through imine linkage. The presence of polymer, therefore, does not hinder imine COF formation.

Furthermore, an absorption peak characteristic of the C=O bond in PDMAEMA is detected at 1721 cm^−1^ and 1723 cm^−1^ in the spectra of *I‐COF‐Poly‐0.5* and *I‐COF‐Poly‐0.1*, respectively. As *I‐COF‐Poly‐0.5* is the COF with the highest polymer concentration, its spectrum is distinguished from other COFs by the presence of absorption bands at 2772 and 2823 cm^−1^, characteristic of the elongation of the C—H bond of the N(CH_3_)_2_ group of PDMAEMA.

#### 3.1.3 Crystallinity

The simulated diffractogram of *I‐COF* exhibits four main peaks (Figure 2). The most intense peak, at 3.18°, corresponds to the (100) plane, while additional peaks are observed at 7.65° and 19.70°, associated with the (210) and (001) planes, respectively. Finally, the peak at 13.64° corresponds to three closely spaced diffraction planes: (300), (330), and (030). These four peaks are also present in the diffractograms of *I‐COF‐80 °C*, *I‐COF‐Poly‐0.01*, and *I‐COF‐Poly‐0.1*, confirming that the synthesized COFs match the expected structure. The 2*θ* angles and interplanar distances corresponding to these peaks are detailed in Table S2, Supporting Information. In contrast, the diffractogram of *I‐COF*, obtained *via* solvothermal synthesis, displays only the first two characteristic peaks of the (100) and (210) planes, while the remaining peaks appear less well‐defined. Furthermore, the comparison between the experimental PXRD of *I‐COF* and the simulated PXRD of AA and AB stacking of *I‐COF* showed that AA stacking appears to be the most energetically favorable model (Figure S8, Supporting Information).

Additional peaks, labeled with a star (*), are also observed on the diffractograms of *I‐COF‐80 °C*, *I‐COF‐Poly‐0.01*, and *I‐COF‐Poly‐0.1* (Figure 2). Their appearance may be attributed to partial staircase stacking of the COF layers. Since COFs consist of stacked layers held together by weak noncovalent interactions, various geometric configurations—such as eclipsed, staggered, or oblong arrangements—can occur during stacking.^[^ ^46^, ^47^ ^]^ These stacking irregularities can result in the splitting of diffraction peaks. It should be noted that this duplication is not found in *I‐COF*, probably indicating better crystallinity and more regular stacking.

Broad diffraction peaks are also observed, suggesting the possible formation of a semicrystalline material with limited short‐range order. For *I‐COF‐Poly‐0.5*, however, only polymer‐related signals are detected, with broad peaks characteristic of PDMAEMA appearing at 7.5° and 17.5°.^[^ ^48^ ^]^ This result aligns with the synthesis conditions, as the polymer accounts for 78 wt% of the total precursor mass initially introduced. While the presence of polymer does not appear to completely inhibit COF crystallization, the high polymer content in *I‐COF‐Poly‐0.5* makes it difficult to confirm COF crystallite formation in this case. Additionally, the use of a growth‐blocking agent seems to increase plane‐stacking defects, as evidenced by the presence of split peaks.

#### 3.1.4 Morphology

**Figure 3:**
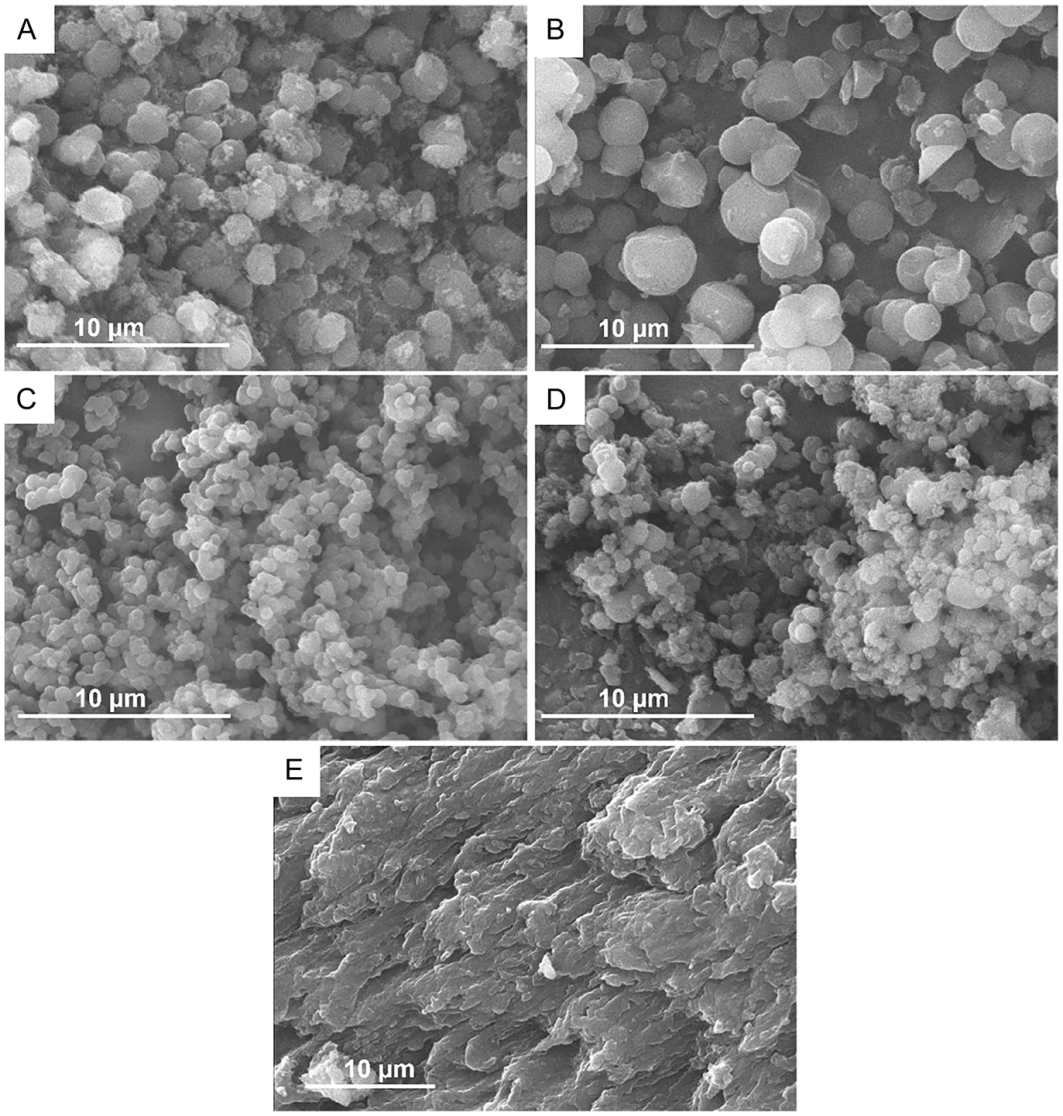
SEM images of COFs synthesized in dry state without a blocking agent: A) *I‐COF* and B) *I‐COF‐80 °C*; and in the presence of a macromolecular blocking agent: C) *I‐COF‐Poly‐0.01*, D) *I‐COF‐Poly‐0.1*, and E) *I‐COF‐Poly‐0.5*.

The morphology of the precipitates was compared by SEM (**Figure** **3**).

First, COFs synthesized without a blocking agent were compared (Figure 3A,B). Under mild conditions (*I‐COF‐80 °C*), smooth spherical particles with diameters ranging from 1.5 to 3.8 μm were observed (Figure 3B). Similarly, solvothermal synthesis (*I‐COF*) also yielded spherical particles, but with smaller diameters (1–2 μm) and a rougher surface texture, appearing to be covered with small aggregates (Figure 3A). This suggests that synthesis under mild conditions can already limit the aggregation of COF particles as well as their aggregation rate, thereby allowing the formation of larger COF particles.

In the presence of a macromolecular blocking agent, the resulting morphologies differ depending on the polymer ratio introduced. At a low polymer ratio (*I‐COF‐Poly‐0.01*), the COF particles remain spherical and uniform in size, with diameters ranging from 550 to 750 nm (Figure 3C). With an intermediate polymer ratio (*I‐COF‐Poly‐0.1*), spherical particles are still present but exhibit a wider size distribution (400–800 nm) and a higher degree of aggregation compared to *I‐COF‐Poly‐0.01* (Figure 3D). Finally, for the highest polymer ratio (*I‐COF‐Poly‐0.5*), small particles appear embedded within a polymer film, making it difficult to clearly distinguish individual COF particles (Figure 3E). Overall, the presence of a macromolecular blocking agent during synthesis leads to smaller COF particles compared to syntheses without a blocking agent. This supports the hypothesis that the blocking agent limits COF growth. However, when the polymer content is too high, polymer chains grafted onto the particles may become entangled, promoting particle aggregation in the dry state.

Nitrogen sorption measurements were performed to characterize the specific surface area and assess the microporosity of the different COFs (Figure S9, Supporting Information). All samples exhibit a characteristic type‐IV mesoporous N_2_ isotherm, with the exception of sample *I‐COF‐80 °C*, which exhibits a type‐V isotherm. None of the samples exhibits microporosity. The (BET) brunauer‐emmett‐teller surface areas are reported in **Table** **2**.

**Table 2:** Textural properties of the different COFs determined by N_2_ adsorption–desorption at 77 K from the BET and (BJH)barrett‐joyner‐halenda methods.

Sample	S BET [m^2^ g^−1^]	Total pore volume [cm^3^ g^−1^]	Average pore size [nm]
*I‐COF*	781	1.28	2.5
*I‐COF‐80 °C*	318	9.75	3.7
*I‐COF‐Poly‐0.01*	29	0.37	8.1
*I‐COF‐Poly‐0.1*	34	1.22	4.0
*I‐COF‐Poly‐0.5*	17	0.76	4.2

The *I‐COF* synthesized with standard synthetic conditions presents a high surface area of 781 m^2^ g^−1^, which decreases to 318 m^2^ g^−1^ when low temperature is used for *I‐COF‐80 °C.* The samples containing a macromolecular growth‐blocking agent present a strong decrease in the surface area to around 30 m^2^ g^−1^, which may be related to the low accessibility of the COF core by N_2_ molecules due to the collapse of the polymer chains on the surface of the COF particles in the dry state. Furthermore, the addition of a blocking agent appears to have an effect on the size of the mesopores, with average pore sizes close to 4 nm compared to 2.5 nm for *I‐COF* (Table 2), with a broad size distribution from 2 to 80 nm (Figure S9, Supporting Information). This broad distribution of mesopores to macropores is important for photocatalysis applications to improve the specific surface area and allow species to move to the active sites present in the pores. Improving microporosity would promote the formation of a hierarchical pore network, thereby improving the photocatalytic performance of COF particles.^[^ ^49^ ^]^

#### 3.1.5 Behavior and Stability in Water

**Figure 4:**
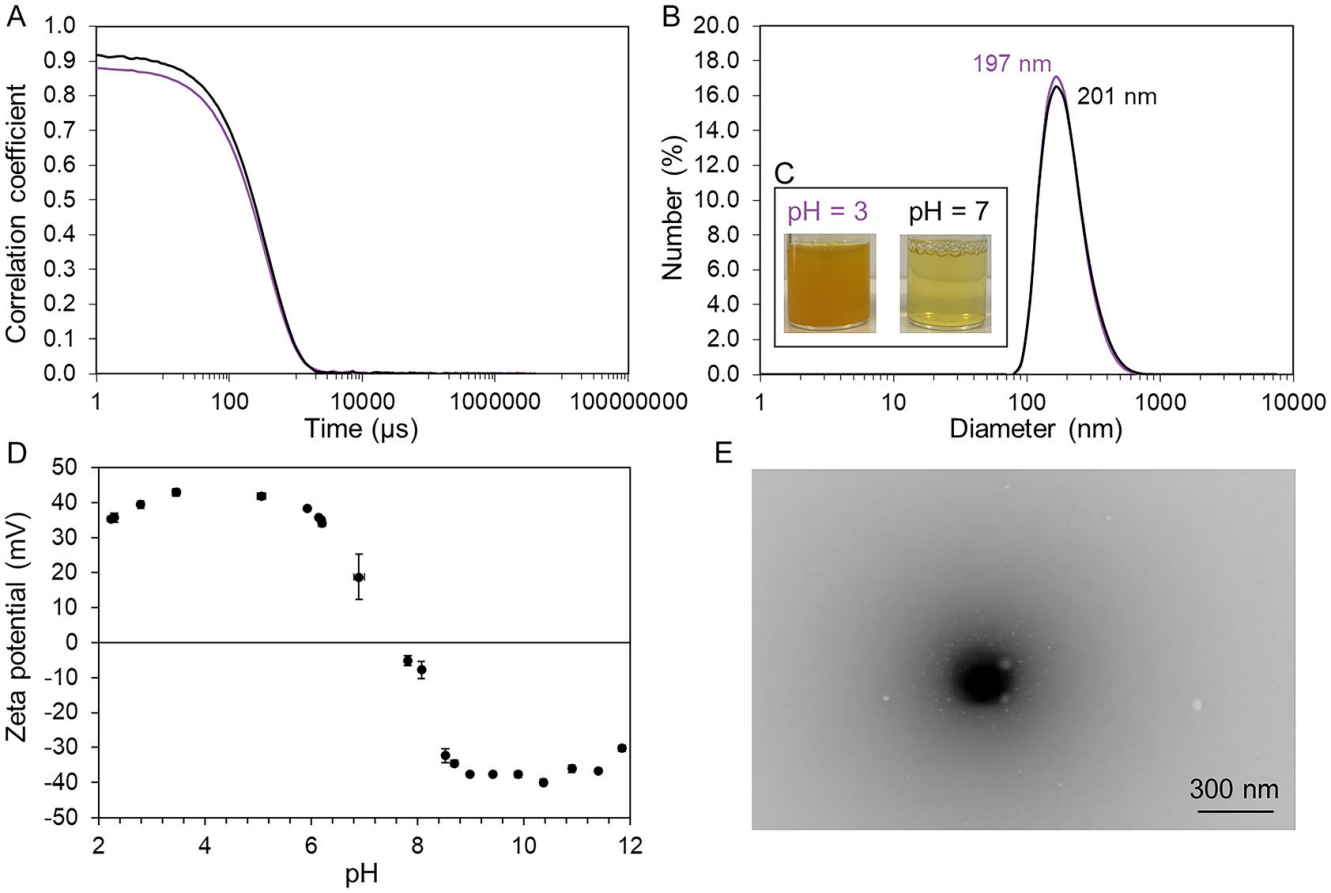
A–C) DLS, D) zeta potential measurement, and E) STEM of *I‐COF‐Poly‐0.5* redispersed in water at pH3 (purple) and pH7 (black) @ 0.05 g L^−1^. A) Correlograms. B) Number size distribution. C) Photographs of dispersions prepared using the method described in Figure S1, Supporting Information. D) Zeta potential versus pH. E) STEM images of *I‐COF‐Poly‐0.5* in dry state.

Since *I‐COF‐Poly‐0.5* was the only sample to remain stable beyond the 8‐hour reaction time and present the lower bandgap value, its solubility in aqueous media was tested to develop water‐stable colloidal dispersions suitable for photocatalytic hydrogen production (Figure S1, Supporting Information). In addition, two pH values were considered to potentially improve the COF particles’ solubility in water. As PDMAEMA has a *pKa* of 6.5,^[^ ^50^ ^]^ its polymer chains remain uncharged at pH 7, while at pH 3, they are protonated and exist in their polyelectrolyte form as quaternary ammonium species. This ionization could induce electrostatic repulsions between polymer chains and colloidal particles,^[^ ^51^ ^]^ potentially promoting aggregate dissociation and enhancing the stabilization of COF particles in solution. The redispersion of *I‐COF‐Poly‐0.5* was successful at both tested pH values (**Figure** **4**C insert).

DLS analysis confirmed that both dispersions exhibit correlograms characteristic of colloidal behavior (Figure 4A), demonstrating that the COF particles were stabilized by the polymer chains. The measured hydrodynamic diameters were nearly identical at both pH values, 197 nm at pH 3 and 201 nm at pH 7 (Figure 4B), suggesting that acidic conditions do not significantly enhance aggregate dissociation or that the measured sizes correspond to the original colloidal COF particles. The STEM analysis confirms a diameter of the COF around 200 nm (Figure 4E). Herein, we used an oligomer with a low molecular weight of 6,000 g.mol^−1^, representing ≈35 monomer units, *i.e.*, a maximum length of 9 nm if fully stretched in a good solvent which do not significantly modify the whole diameter of the colloidal COF. It is worth noting that these polymer chains collapse in a bad solvent, *i.e.*, dry state in air or vacuum media of the STEM chamber, to less than 2 nm.^[^ ^52^ ^]^ This is the main reason why the polymer layer cannot be directly observed under STEM conditions. Nevertheless, indirect evidence of a polymer layer can be found in the observation of COF surface degradation, *i.e.*, small white dots, due to the organic amorphous state of the PDMAEMA chains (Figure 4E).

Concerning the pH‐sensitiveness of the PDMAEMA chains and therefore of the colloidal *I‐COF‐Poly‐0.5*, zeta potential was measured versus pH (Figure 4D). As previously mentioned, the PDMAEMA monomer unit has a pKa value around 6.5. In Figure 4D, we can definitively demonstrate the presence of the polymer chains with a positive value around +40 mV up to pH 7, *i.e.*, characteristic of a positively charged, highly stable colloid. When the PDMAEMA pKa is reached between pH 7 and 8, colloidal stability drops with an isoelectric point at pH 7.6 due to the screening of the electrostatic repulsions of the PDMAEMA. Then the values decrease and become negative around ζ = −40 mV for pH higher than 8.5 and characteristic of a colloidal stable system. This behavior is due to the ionization of phenolic moieties of the *I‐COF‐Poly‐0.5*, *i.e.*, similar to the 1,3‐dihydroxybenzene low acid with double pKa values around 9.5 and 11.3, which in the presence of the polybase PDMAEMA is strongly deprotonated.

A notable color difference was also observed between the two dispersions (Figure 4C), likely due to protonation of the PDMAEMA's amine and COF's imine functionalities at acidic pH, which modifies its optical properties.^[^ ^13^, ^53^ ^]^ Theoretical studies have shown that protonation of imine bonds leads to a flatter COF lattice conformation, increasing conjugation.^[^ ^54^ ^]^ This enhanced conjugation could contribute to a reduced bandgap, potentially improving the COF's photocatalytic performance.

### 3.2 Hydrogen Production by Photocatalysis

COFs can absorb light radiation to drive photocatalytic hydrogen production. Upon light absorption, an electron is excited from the highest occupied molecular orbital (HOMO) to the lowest unoccupied molecular orbital (LUMO) of the COF, leaving behind a hole in the HOMO. To enhance charge separation, a metallic cocatalyst is usually added to the system. The photoexcited electron is transferred to the cocatalyst, which facilitates proton reduction to generate H_2_. To prevent COF photodegradation and regenerate the holes in the HOMO, a SED is also added as an electron source. This sacrificial agent replaces the water oxidation half‐reaction, which is both kinetically and thermodynamically difficult to achieve.^[^ ^55^ ^]^ As the photocatalytic process progresses, the sacrificial agent undergoes irreversible degradation and is consequently consumed. To determine whether modifying a COF into a colloidal form affects its photocatalytic properties, *I‐COF‐Poly‐0.5* was tested for hydrogen production in ultrapure water. Several experimental parameters were investigated: (i) *I‐COF‐Poly‐0.5* photocatalyst concentration: 0.05 to 1.0 g.L^−1^; (ii) type of SED: ascorbic acid, sodium ascorbate, and triethanolamine (TEOA); and (iii) cocatalyst salt: platinum salt (H_2_PtCl_6_ and K_2_PtCl_6_) and silver salt (AgNO_3_).

#### 3.2.1 Impact of Photocatalyst Concentration

**Figure 5:**
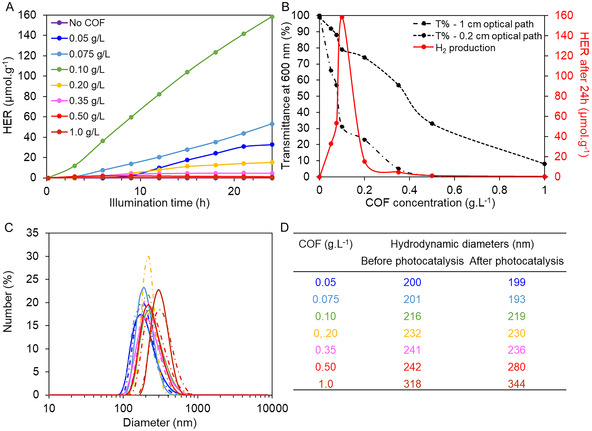
A) Hydrogen production over time at different photocatalyst concentration: no COF (purple), 0.05 g.L^−1^ (dark blue), 0.075 g.L^−1^ (light blue), 0.10 g.L^−1^ (light green), 0.20 g.L^−1^ (yellow), 0.35 g.L^−1^ (pink), 0.50 g.L^−1^ (red), and 1.0 g.L^−1^ (brown). B) Hydrogen produced after 24 h (red curve) versus solution transmittance before photocatalysis in 1 cm (solid black line) and 0.2 cm (dotted black line) cuvettes. C) Size distributions before (solid line) and after (dash‐dotted line) photocatalysis. D) Hydrodynamic diameters from (C).

The effect of photocatalyst concentration on hydrogen production was investigated over a range of 0.05–1.0 g.L^−1^ of *I‐COF‐Poly‐0.5* (**Figure** **5**).

These concentrations were calculated based solely on the theoretical mass of the COF, excluding the polymer component of *I‐COF‐Poly‐0.5*, which accounts for ≈78 wt% of the material. Sodium ascorbate (0.1 mol.L^−1^) was used as the SED, and hexachloroplatinic acid (H_2_PtCl_6_, 10^−4^ mol.L^−1^) served as the cocatalyst. Figure 5A illustrates the evolution of hydrogen production over time as a function of photocatalyst concentration. From 0 to 0.1 g.L^−1^, hydrogen production increases with photocatalyst concentration, indicating that higher COF concentrations initially enhance photocatalytic performance. However, beyond 0.1 g.L^−1^, H_2_ production decreases as COF concentration increases. This decline can be attributed to a significant reduction in solution transmittance at concentrations exceeding 0.1 g.L^−1^. Indeed, Figure 5B shows that transmittance decreases sharply between 0 and 0.1 g.L^−1^, then more gradually from 0.1 to 0.4 g.L^−1^, approaching zero between 0.5 and 1.0 g.L^−1^. This trend can be explained by the light scattering phenomenon that occurs in colloidal dispersions. The presence of particles leads to scattering known as the “Tyndall effect”, whose variation is given by the equation: $A_{\text{diff}}=\;C^{\text{ste}}\times \frac{1}{\lambda^{n}}$ where *A*_diff_ represents absorbance due to scattering and *n* varying from 2 to 4.^[^ ^56^ ^]^ If the COF concentration is too high, light scattering is high, reducing the deep penetration of light and then hampering the photocatalysis process. Therefore, a balance must be reached between a sufficiently high photocatalyst concentration and a solution that transmits deeper into the light to ensure optimal performance. According to Figure 5B, a COF concentration of 0.1 g.L^−1^ appears to be the most suitable for achieving optimal photocatalytic performance with this system.

DLS analysis revealed that all solutions exhibited colloidal behavior both before and after photocatalysis, regardless of *I‐COF‐Poly‐0.5* concentration (Figure S, Supporting Information). The hydrodynamic particle diameters remained more or less similar across all concentrations from 0.05 to 0.50 g.L^−1^ (Figure 5C,D). At 1.0 g.L^−1^ COF, the measured diameters were larger, probably due to excessive steric hindrance leading to particle aggregation.

#### 3.2.2 Impact of the Photocatalyst Size

**Figure 6:**
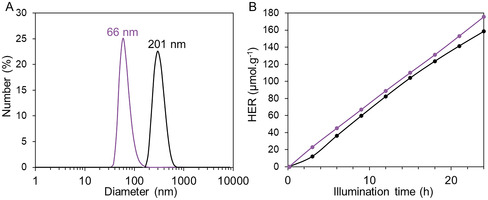
A) Size distribution of *I‐COF‐Poly‐0.5* synthesized using PDMAEMA of 6,000 g.mol^−1^ (black) and 3,200 g.mol^−1^ (purple) @ 0.05 g L^−1^. B) Hydrogen production over time as a function of photocatalyst size with sodium ascorbate as SED and H_2_PtCl_6_ as cocatalyst: diameter of 216 nm in black and 66 nm in purple.

The molecular weight of the polymer used as a growth‐blocking agent significantly influenced the size of the resulting colloidal COF particles (**Figure** **6**).

Using a 6,000 g.mol^−1^ PDMAEMA leads to COF particles with an average diameter of 201 nm, compared to just 66 nm for a molecular weight of 3,200 g.mol^−1^ (Figure 6A). The difference in size is attributed to the higher number of solvophilic groups present in the longer polymer chain, which enhances its overall solvophilicity in the synthesis media and allows more COF units to be stabilized before self‐assembling into colloidal structures. In contrast, a shorter polymer reaches the COF‐modified amphiphilic balance faster, triggering earlier self‐assembly and resulting in smaller particles. This phenomenon is well‐known for emulsion polymerization by similar PISA process.^[^ ^34^ ^]^

Moreover, the difference of colloidal COF size impacts the photocatalytic performance of the system (Figure 6B), with a hydrogen production of 158 μmol.g^−1^ for the ≈200 nm particles in diameter after 24 h, compared to 176 μmol.g^−1^ for ≈70 nm particles, *i.e.*, corresponding to an increase of more than 10%. The higher H_2_ yield observed with smaller COF particles can be attributed to their larger specific surface area, providing more active sites for interaction with the platinum cocatalyst. Additionally, the reduced particle size facilitates faster charge transfer, thereby limiting exciton recombination and enhancing overall photocatalytic efficiency.^[^ ^29^ ^]^

#### 3.2.3 Impact of the SED

**Figure 7:**
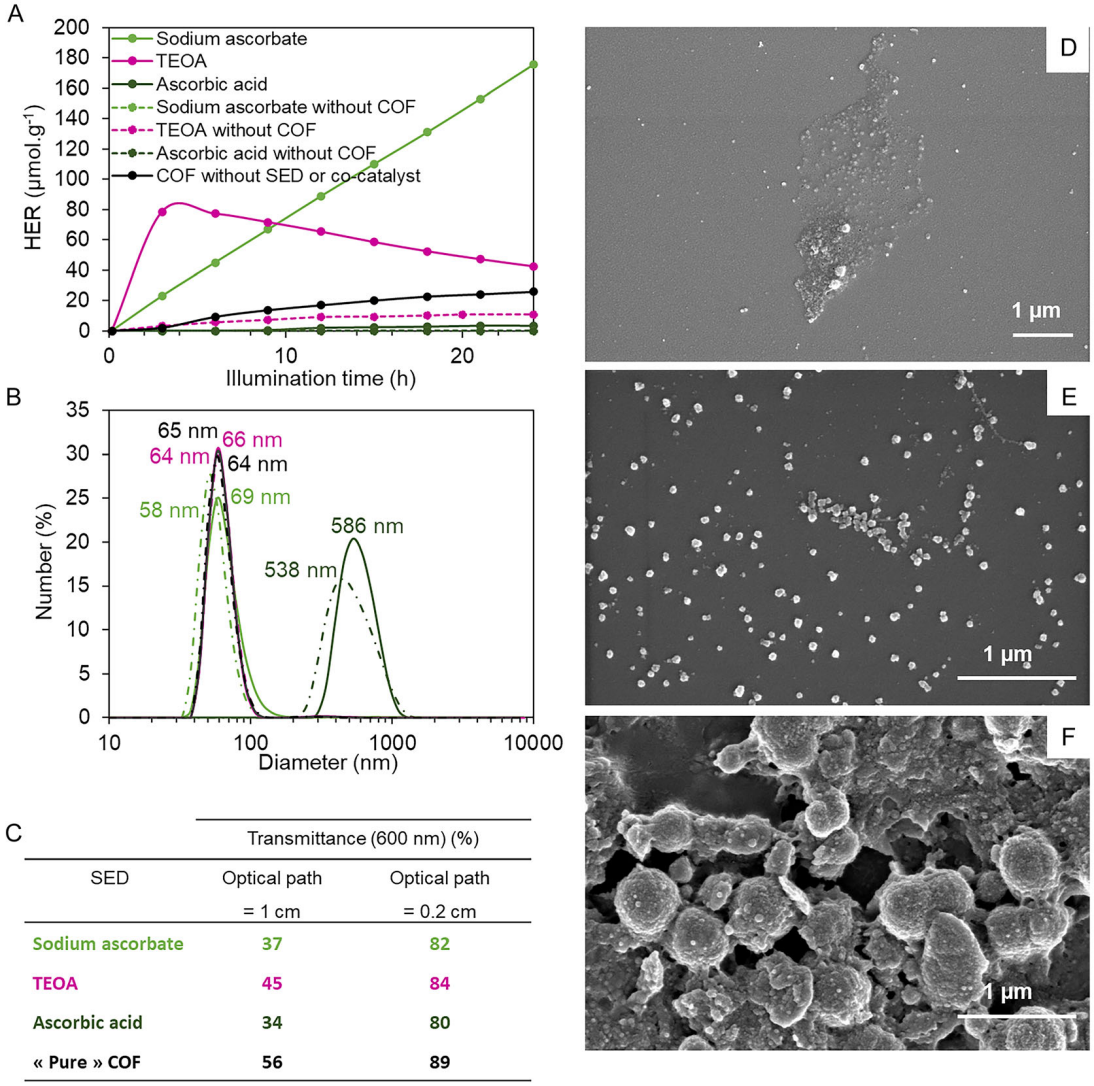
A) Hydrogen production over time with different SEDs (solid lines): sodium ascorbate (light green), ascorbic acid (dark green), and TEOA (purple). Control experiments without photocatalysts (dotted lines), and without SED and cocatalyst (black curve). B) Size distribution before (solid lines) and after (dash‐dotted lines) photocatalysis. C) Transmittances of the solutions tested in photocatalysis. D–F) SEM images of the solutions deposited on silicon wafer before photocatalysis for (D) sodium ascorbate, (E) TEOA, and (F) ascorbic acid.

The influence of the SED on hydrogen production was investigated by testing three benchmarked SEDs: ascorbic acid,^[^ ^13^ ^]^ sodium ascorbate,^[^ ^12^ ^]^ and TEOA^[^ ^18^ ^]^ (**Figure** **7**). To ensure consistency across experiments, each donor was introduced at a fixed concentration of 0.1 mol.L^−1^, while the concentration of *I‐COF‐Poly‐0.5* with around 70 nm in diameter, *i.e.*, with the highest efficiency (see Section 3.2.2), was set at 0.1 mol.L^−1^, and the platinum salt H_2_PtCl_6_ (10^−4^ mol.L^−1^) was used as the cocatalyst.

Figure 7A illustrates the evolution of hydrogen production over time, as a function of the sacrificial agent. The results show that hydrogen is produced when using sodium ascorbate or TEOA, whereas no hydrogen is detected in the presence of ascorbic acid. This disparity can be attributed to differences in pH. While the sodium ascorbate and TEOA solutions are slightly basic (pH = 7.5), the ascorbic acid solution is acidic (pH = 3). At low pH, although protons are more abundant, the imine bonds in *I‐COF‐Poly‐0.5* may undergo protonation, as discussed before, which enhances conjugation. However, these bonds are also susceptible to hydrolysis, potentially compromising the COF network. In contrast, at neutral or basic pH, imine bonds are more stable, allowing the COF structure to remain intact. Additionally, hydroxyl groups within the COF framework can be protonated under acidic conditions, leading to water elimination.^[^ ^57^ ^]^ Since these hydroxyl groups contribute to intramolecular hydrogen bonding (O—H····N=C), which reinforces the COF's stability and crystallinity,^[^ ^39^ ^]^ their protonation may further destabilize the network. Overall, neutral pH conditions appear to be more favorable for hydrogen production with *I‐COF‐Poly‐0.5*.

Despite both sodium ascorbate and TEOA facilitating hydrogen evolution at slightly basic pH, their efficiency differs significantly (Figure 7A). With sodium ascorbate, hydrogen production follows a steady, linear trend at ≈7 μmol.g^−1^.h^−1^. In contrast, TEOA initially drives a much faster hydrogen evolution rate of around 26 μmol.g^−1^.h^−1^ during the first three hours. However, this rapid production is followed by a faster deactivation of the photocatalytic system,^[^ ^12^, ^55^ ^]^ as the detected H_2_ concentration declines progressively over 21 h. This may happen either because hydrogen production decreases over time or because it drops to zero production after 5 h, leading to less hydrogen detection due to repeated headspace sampling. According to the literature, TEOA is generally used for short irradiation times (under 6 h). For prolonged illumination, sodium ascorbate appears to be the most suitable sacrificial agent for the *I‐COF‐Poly‐0.5*/Pt system.

Control experiments conducted without a photocatalyst resulted in negligible H_2_ production (Figure 7A), confirming that the observed catalytic activity originates from *I‐COF‐Poly‐0.5*. Additionally, a test was performed in the presence of sodium ascorbate, H_2_PtCl_6_, and COF, but under dark conditions. This experiment yielded no hydrogen production, demonstrating that photocatalytic activity is exclusively light‐driven in the presence of colloidal COF. The intrinsic photocatalytic activity of the “pure” COF was also evaluated in the absence of any SED or platinum cocatalyst (Figure 7A). Under these conditions, *I‐COF‐Poly‐0.5* still exhibited a non‐negligible hydrogen evolution, reaching 25 μmol.g^−1^ after 24 h, which corresponds to ≈7 times less of the total H_2_ produced when sodium ascorbate and platinum are present. This unexpected H_2_ production in the absence of an external SED could be attributed to the polymer used as a blocking agent during the synthesis of the colloidal COF, which may itself act as a sacrificial agent. Notably, PDMAEMA contains ester functionalities, and previous work by Uekert et al. demonstrated that polyesters (e.g., PET) can participate in photocatalysis as sacrificial donors.^[^ ^58^ ^]^

DLS analysis showed that all three solutions exhibited colloidal behavior both before and after photocatalysis (Figure S11, Supporting Information). The hydrodynamic diameters of the particles remained similar before and after photocatalysis but varied depending on the electron donor used (Figure 7B). Notably, systems containing sodium ascorbate and TEOA displayed a single population of particles with diameters around 70 nm, consistent with the size of COFs dispersed in ultrapure water. In contrast, the system containing ascorbic acid exhibited significantly larger particles, ≈550 nm in diameter. The increased particle size in the presence of ascorbic acid could be due to aggregate formation at acidic pH, whereas sodium ascorbate and TEOA contribute to a slightly basic pH, preventing aggregation. This hypothesis is further supported by SEM images. While the particles observed in the sodium ascorbate and TEOA systems measure around 50–70 nm in diameter (Figure 7D,E), aggregates ranging from 400 to 600 nm are detected in the ascorbic acid system (Figure 7F). For sodium ascorbate and TEOA, the particle sizes measured by SEM are smaller than those obtained by DLS (≈60 nm versus 70 nm in solution), a phenomenon characteristic of the collapse of PDMAEMA chains in air. In this case, the apparent aggregation observed in SEM probably results from drying effects during sample preparation. However, in the presence of ascorbic acid, significantly larger aggregates are observed, suggesting that COF particles may become trapped within these structures. This aggregation could also explain the negligible hydrogen production measured for the ascorbic acid system. If COF particles are embedded within aggregates with limited surface access for the platinum salt, electron transfer from COF to Pt cannot occur efficiently, restricting proton reduction to the outer surface of the aggregates.

Finally, the transmittance of solutions prepared with different electron donors was compared (Figure 7C) to assess whether the choice of SED influences solution transmittance and, consequently, the absorption properties of the photocatalytic system. At 600 nm (mid‐visible range), all three sacrificial agents yielded similar transmittance values: ≈35%–40% for the 1 cm cuvette and 80%–85% for the 0.2 cm cuvette. While TEOA provided slightly better transmittance, the choice of sacrificial agent does not appear to significantly impact the optical properties of the system. Furthermore, solution transmittance declines rapidly with increasing optical path length, dropping to just around 40% at 1 cm. This suggests that using a large irradiated solution volume in the photocatalytic cell may not necessarily enhance photocatalytic performance.

#### 3.2.4 Impact of the Cocatalyst

**Figure 8:**
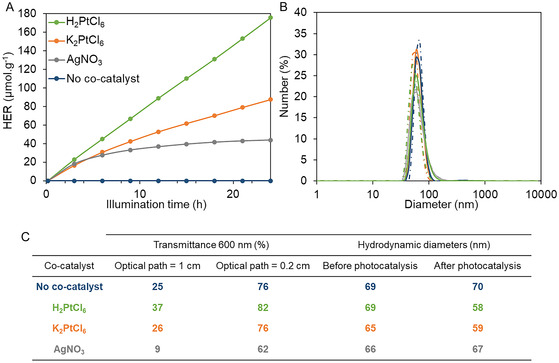
A) Hydrogen production over time as a function of the different cocatalysts used: H_2_PtCl_6_ (light green), K_2_PtCl_6_ (orange), AgNO_3_ (gray), without cocatalyst (dark blue). B) Size distribution of the solutions tested in photocatalysis as a function of the cocatalyst. Solid lines correspond to the solution before photocatalysis, and dash‐dotted lines after photocatalysis. C) Table presenting the transmittances of the solutions tested in photocatalysis and hydrodynamic diameters of (B).

Finally, the influence of the cocatalyst in the photocatalytic system was examined using different metal salts: H_2_PtCl_6_, K_2_PtCl_6_, and AgNO_3_ (**Figure** **8**). To ensure consistency, all salts were introduced at the same concentration in each experiment. The COF concentration was maintained at 0.1 g.L^−1^, with sodium ascorbate (0.1 mol.L^−1^) as the selected sacrificial agent.

Figure 8A presents the evolution of hydrogen production over time, as a function of the cocatalyst present in the system. In the presence of H_2_PtCl_6_, hydrogen is produced at a consistent rate of ≈7 μmol.g^−1^.h^−1^, indicating a linear increase over time. When K_2_PtCl_6_ is used instead, hydrogen evolution also follows a quasilinear trend, though at a lower rate of about 4 μmol.g^−1^.h^−1^. This suggests that hydrogen generation with H_2_PtCl_6_ is not solely due to the reduction of the platinum salt itself, but also involves the photoreduction of water protons. The difference in hydrogen yield between the two platinum salts can be attributed to the higher proton concentration introduced by H_2_PtCl_6_, which promotes enhanced hydrogen evolution.

On the other hand, when AgNO_3_ is used as the cocatalyst, hydrogen production is initially similar to that observed with platinum during the first six hours. However, it quickly levels off, reaching a much lower rate of around 0.9 μmol.g^−1^.h^−1^ after 6 h of illumination. This behavior may result from a redox reaction between silver nitrate and sodium ascorbate (used as the SED), leading to the formation of colloidal silver nanoparticles.^[^ ^59^ ^]^ Since these nanoparticles are not formed through photoreduction by the COF, they are not necessarily deposited on the COF surface. This lack of surface interaction may hinder the transfer of photoexcited electrons from the COF to the metal cocatalyst. Additionally, the redox reaction between silver nitrate and sodium ascorbate generates a precipitate, which significantly reduces the optical transmission of the solution from 82 to 62% at 600 nm for the platinum and silver salts, respectively, for an optical path length of 0.2 cm (Figure 8C).

A control experiment in the presence of sodium ascorbate and COF, without a cocatalyst, was carried out and led to no H_2_ production after 24 h of illumination, confirming the need to add a metal salt to the system to produce hydrogen.

DLS analysis showed that the solutions all exhibited colloidal behavior both before and after photocatalysis, irrespective of the cocatalyst added (Figure S12, Supporting Information). The hydrodynamic diameters of the particles remained similar before and after photocatalysis for the different solutions, with values ranging between 60 and 70 nm (Figure 8B,C). These results demonstrate that colloidal COF dispersions elaborated by the SAI2COF methodology maintain good stability over time. No precipitation or aggregation was observed after 24 h of photocatalysis, in contrast to previously reported polymeric donor–acceptor (D/A) systems, where nanoparticle aggregation occurred after only 3 h of illumination in the absence of surfactant.^[^ ^60^ ^]^ Similarly, Poly(9,9di‐*n*‐octyl‐9*H*‐fluorene) PFO particles exhibited very limited photocatalytic activity in dispersed form beyond 5 h, unlike their solid‐state film counterparts.^[^ ^42^ ^]^ In addition, the degradation of sodium ascorbate does not appear to disrupt the linear hydrogen evolution rate over 24 h, contrary to findings by Kosco et al.^[^ ^61^ ^]^ and Zhao et al.,^[^ ^29^ ^]^ who reported activity loss with ascorbic acid under similar conditions.

These results are consistent with the work of Wang et al., who showed that COFs incorporating sulfone groups exhibited sustained photocatalytic activity with linear hydrogen production maintained for over 50 h, suggesting the intrinsic stability of sulfonated COFs under prolonged illumination.^[^ ^16^ ^]^ Thomas and coworkers also demonstrated that the chemical functions present in the pores of COFs played a major role in their chemical stability and photocatalytic performance. Indeed, functionalizing the pores with hydrophilic groups coupled with the use of robust chemical bonds, such as quinolone, rather than reversible chemical bonds, such as imines, to bind the elementary building blocks of COFs, enabled them to improve the chemical stability of COFs and produce hydrogen stably for at least 15 days.^[^ ^62^ ^]^

Solutions containing the platinum salt (H_2_PtCl_6_) and the silver salt (AgNO_3_) were analyzed before and after photocatalysis using SP‐ICP‐MS to assess whether metallic nanoparticles had formed. Before light exposure, no metallic nanoparticles were detected in either solution; only dissolved platinum (Figure S13A, Supporting Information) and silver (Figure S13C, Supporting Information) were observed. However, after photocatalysis, metallic nanoparticles were detected in both solutions (Figure S13B,D, Supporting Information). Based on peak intensity and under the assumption that the particles are spherical and composed solely of platinum or silver metals, the average diameters were estimated to be ≈30 nm for platinum and 77 nm for silver (Figure S14, Supporting Information). It is also possible that platinum and silver were partially adsorbed onto the surface of the COF particles, thereby coating the colloids rather than forming freely dispersed nanoparticles.

The absence of metallic nanoparticles prior to illumination confirms that *I‐COF‐Poly‐0.5* is capable of photoreducing metal salts to their corresponding nanoparticles under light irradiation, indicating successful photoinduced electron transfer from the COF to the metal ions. However, while platinum nanoparticles effectively promoted hydrogen evolution, silver nanoparticles showed only limited activity, producing significantly less hydrogen under the same conditions. Two hypotheses may explain this behavior: i) The silver nanoparticles may not be in contact with the COF, possibly due to being freely dispersed or aggregated, which would hinder electron transfer. These particles may have formed through a direct redox reaction between AgNO_3_ and sodium ascorbate, resulting in weak or no interaction with the COF. Interestingly, the particle sizes estimated *via* SP‐ICP‐MS match those obtained by DLS, suggesting either that silver was deposited on the COF surface or that the free silver nanoparticles have similar dimensions to the COF particles, thus preventing resolution of distinct populations in the DLS analysis (Figure 8B). ii) From an electronic perspective, silver's LUMO may be too low to effectively reduce protons, unlike platinum, whose LUMO lies above the reduction potential for proton‐to‐hydrogen conversion. This hypothesis is supported by the work function values: 4.52–4.74 eV for silver and 5.12–5.93 eV for platinum.^[^ ^63^ ^]^ These values suggest that platinum is more suitable as a cocatalyst for hydrogen production in this photocatalytic system.

## 4 Conclusion

The elaboration of colloidal COFs, stabilized by a macromolecular growth‐blocking agent (PDMAEMA) by the SAI2COF method, has enabled to study the influence of the polymer ratio on the size, morphology, crystallinity, and solution stability of colloidal COF particles. These surfactant‐free process shows that using an adjusted PDMAEMA content, *i.e.*, *I‐COF‐Poly‐0.5*, promotes the formation of large and stable colloidal COF particles in solution and prevents their precipitation. Furthermore, the modification of light absorption as a function of polymer ratios reveals that the conjugation of COF networks can be optimized by the use of a blocking agent.

Moreover, we also demonstrated that the size of the colloidal COF can be tuned by the molecular weight of the macromolecular blocking agent, *i.e.*, length of the polymer chain, which also behaves as a stabilizer. Indeed, higher the length, higher the colloidal COF size due to the increase of the solvophilic balance during the self‐assembly‐induced to colloidal COF SAI2COF process. Redispersion tests of *I‐COF‐Poly‐0.5* in water further confirmed its ability to maintain stability in aqueous media, *i.e.*, both at acidic and neutral pH, an essential property for photocatalytic applications, particularly for hydrogen evolution. So, a dual effect is demonstrated by using a functional polymer chain, which control the size and stabilize in colloidal state the COFs synthesized by SAI2COF process.

Photocatalytic tests using colloidal *I‐COF‐Poly‐0.5* particles have shown promising potential for hydrogen production. Several key parameters influencing the system's performance were explored, including the SED, photocatalyst concentration, and type of metal cocatalyst. The study of different SEDs showed that sodium ascorbate was the most efficient for stable and prolonged hydrogen production over 24 h. Sodium ascorbate provides a slightly basic environment favorable to COF stability and structural integrity. In addition, although TEOA enabled rapid hydrogen production at the start of the reaction, the rapid deactivation of the system makes this sacrificial agent less suitable for long‐term reactions. Photocatalyst concentration also has a significant impact on photocatalytic performance. An optimal concentration of 0.1 g.L^−1^ was identified for *I‐COF‐Poly‐0.5*. This concentration offers a good balance between solution transmission, ensuring sufficient deep light penetration and catalytic efficiency. Higher concentrations reduced light transmittance, negatively impacting hydrogen production rates. Regarding the choice of metallic cocatalyst, platinum salts (H_2_PtCl_6_ and K_2_PtCl_6_) were the most efficient in catalyzing proton reduction to hydrogen. Silver nitrate (AgNO_3_), on the other hand, resulted in significantly lower hydrogen production, likely due to the formation of silver nanoparticles that were not properly integrated with the COF system, thereby limiting their catalytic activity.

One of the most remarkable hydrogen evolution rates reported for organic photocatalysts, including COFs, was achieved by Cooper's team, who obtained 33.3 μmol.h^−1^ through nanoscale size reduction using surfactants.^[^ ^29^ ^]^ In comparison, our best system, consisting of 0.1 g.L^−1^ COF, sodium ascorbate as SED, and platinum as cocatalyst, generated a total of 175 μmol.g^−1^ after 24 h of reaction, corresponding to an average rate of about 7.3 μmol.h^−1^. The need for further research to better improve the performance and sustainability of COF‐based photocatalysts, SED origin, and efficient noble‐free catalysts are the next evolutions. Positively, the present results are an exciting step paving the development of surfactant‐free and highly stable, efficient photocatalytic colloidal COF systems for clean energy production in the longer term water photolysis.

## Supporting Information

Supporting Information is available from the Wiley Online Library or from the author. Schematic of the redispersing strategies for colloidal COF in water, instrumentation and acquisition parameters set for SP‐ICP‐MS analyses, emission spectrum of the LED lamp used for photocatalysis tests, photo of the photocatalysis setup, photos of reaction mixtures of the colloidal COFs, normalized UV‐visible spectra of the kinetic follow‐up for the different syntheses, infrared spectra of COFs and their precursors zoomed in from 1000 to 2000 cm^−1^, interplanar distances *d* and *2θ* values associated with the peaks identified on the diffractograms in Figure 2, correlograms of the solutions tested in photocatalysis, raw SP‐ICP‐MS signals and average masses of platinum and silver detected per particles by SP‐ICP‐MS.

## Conflict of Interest

The authors declare no conflict of interest.

## Supporting information

Supplementary Material

## Data Availability

The data that support the findings of this study are available in the supplementary material of this article.
