# Supplementary material for: Photogeneration of Hydrogen in Water via Self‐Assembly‐Induced Colloidal Covalent Organic Framework Particles
Source: Small Sci. 2026 Mar 6;6(3):e202500450. doi: 10.1002/smsc.202500450 (PMC13097526; doi:10.1002/smsc.202500450)
Supplement: Supplementary file 1 — Supplementary Material [file SMSC-6-e202500450-s001.pdf]

Supporting Information

Photo-generation of Hydrogen *via* colloidal covalent organic framework particles in water

*Axelle Larrieu, Sabuj Kanti Das, Aurelien Viterisi, and Laurent Billon **

A. Larrieu, S. K. Das, A. Viterisi, L. Billon
Bio-inspired Materials Group: Functionalities & Self-assembly
Institut des Sciences Analytiques et Physico-chimie pour l'environnement et les matériaux
Universite de Pau et des Pays de l'Adour
64000 Pau, France
E-mail: laurent.billon@univ-pau.fr

Figure S1. Schematic of the proposed strategy for redispersing colloidal COF in water. 3
Figure S2. Emission spectrum of the LED lamp (black) used in photocatalysis tests and comparison with the solar spectrum (yellow). 4
Figure S3. Photo of the set-up used for photocatalysis tests. 5
Figure S4. Photos of reaction mixtures after 24h. 5
Figure S5. UV-visible spectra of the reaction mixture normalized on the amine precursor peak at 290 nm, at different times for syntheses (A) *I-COF-80°C*, (B) *I-COF-Poly-0.01*, (C) *I-COF-Poly-0.1* and (D) *I-COF-Poly-0.5*. 6
Figure S6. Band gap of IPREM-COF (black) and *I-COF-Poly-0.5* (purple). 6
Figure S7. Infrared spectra of COFs and their precursors over the full analytical range (A) and zoomed in from 1000 to 2000 cm^{-1} (B). 7
Figure S8. Comparison between experimental PXRD (black) and simulated PXRD of AA (pink) and AB (purple) stacking of *I-COF*. 8
Figure S9. (A) N_2 sorption isotherms and (B) zoom in the 0-900 cm^3/g range. (C) Pore size distribution calculated from the adsorption branch of isotherms by the BJH model and (D) zoom in the 0 to 30 cm^3/g range. 9
Figure S10. Correlograms of the solutions tested in photocatalysis as a function of photocatalyst concentration: 0.05 $g.L^{-1}$ (dark blue), 0.075 $g.L^{-1}$ (light blue), 0.10 $g.L^{-1}$ (light green), 0.20 $g.L^{-1}$ (yellow), 0.35 $g.L^{-1}$ (pink), 0.50 $g.L^{-1}$ (red), and 1.0 $g.L^{-1}$ (brown). Solid lines correspond to the solution before photocatalysis, and dash-dotted lines after photocatalysis. 10
Figure S11. Correlograms of the solutions tested in photocatalysis as a function of the SED: sodium ascorbate (light green), ascorbic acid (dark green), and TEOA (purple). Solid lines correspond to the solution before photocatalysis, and dash-dotted lines after photocatalysis. 11
Figure S12. Correlograms of the solutions tested in photocatalysis as a function of the different co-catalysts used: H_2PtCl_6 (light green), K_2PtCl_6 (orange), $AgNO_3$ (grey), without co-catalyst (dark blue). Solid lines correspond to the solution before photocatalysis, while dash-dotted lines correspond to after photocatalysis. 11
Figure S13. Raw SP-ICP-MS signals as a function of time for solutions tested under photocatalysis: in the presence of H_2PtCl_6 co-catalyst (A) before and (B) after photocatalysis; and in the presence of $AgNO_3$ (C) before and (D) after photocatalysis. 12
Figure S14. Average mass of (A) platinum and (B) silver detected per particles by SP-ICP-MS. 13
Table S1. Instrumentation and acquisition parameters set for SP-ICP-MS analyses. 3

Table S2. Interplanar distances *d* and *2θ* values associated with the peaks identified on the diffractograms in Fig. 2.. 8
Table S3. Textural properties of the different COFs determined by N_2 adsorption–desorption at 77 K from the BET and BJH methods. .... **Error! Bookmark not defined.**

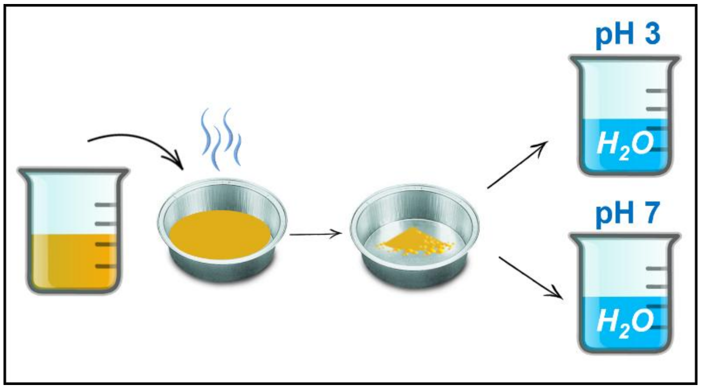

Figure S1. Schematic of the proposed strategy for redispersing colloidal COF in water.

Table S1. Instrumentation and acquisition parameters set for SP-ICP-MS analyses.

**Instrumental parameters**	
RF power	1600 W
Plasma gas flow rate (Ar)	16 L.min^{-1}
Nebulizer	1.1 mL.min^{-1}
Sample uptake rate	0.315 mL.min^{-1}
**Data acquisition parameters**	
Dwell time	100 µs
Total acquisition time	100 s
Isotope monitoring	^{107}Ag, ^{195}Pt

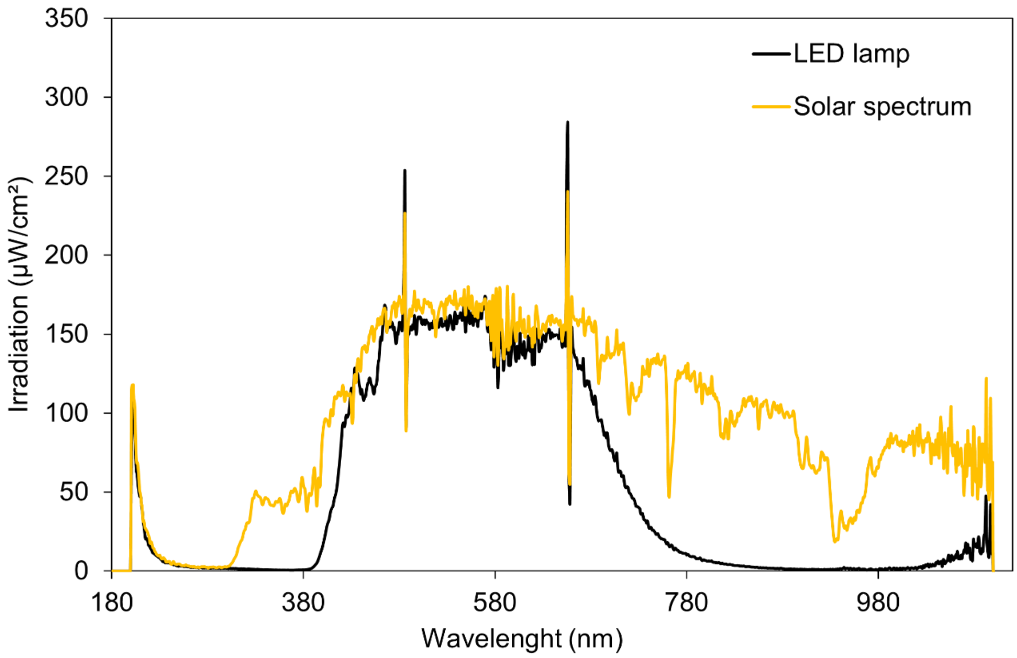

Figure S2. Emission spectrum of the LED lamp (black) used in photocatalysis tests and comparison with the solar spectrum (yellow).

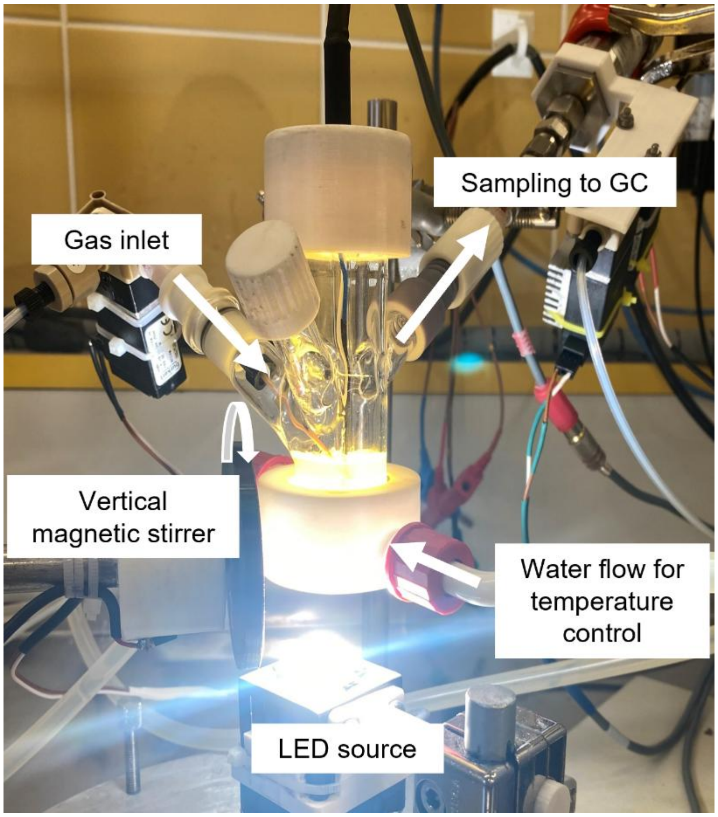

Figure S3. Photo of the set-up used for photocatalysis tests.

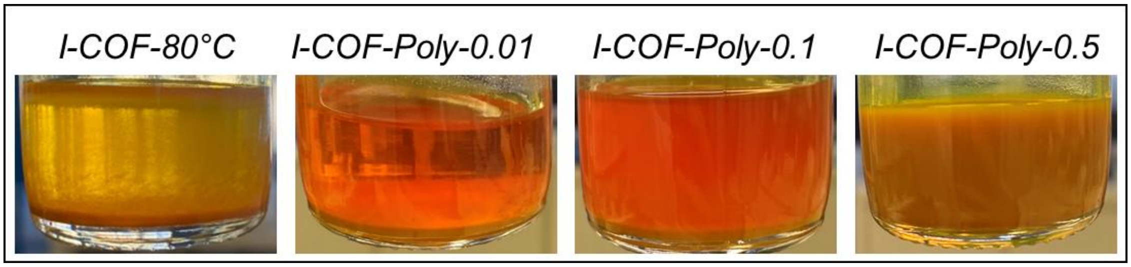

Figure S4. Photos of reaction mixtures after 24h.

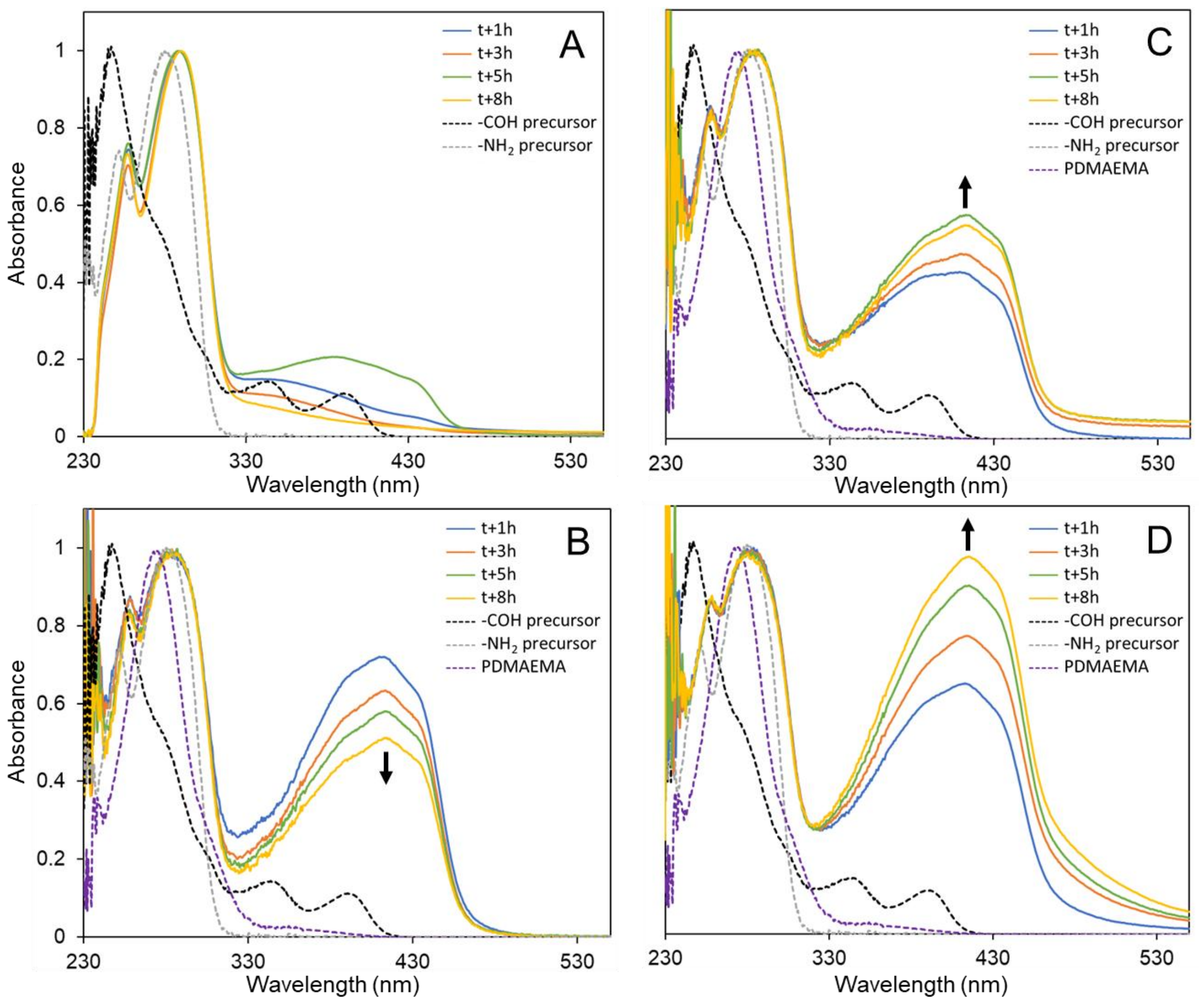

Figure S5. UV-visible spectra of the reaction mixture normalized on the amine precursor peak at 290 nm, at different times for syntheses (A) *I-COF-80°C*, (B) *I-COF-Poly-0.01*, (C) *I-COF-Poly-0.1* and (D) *I-COF-Poly-0.5*.

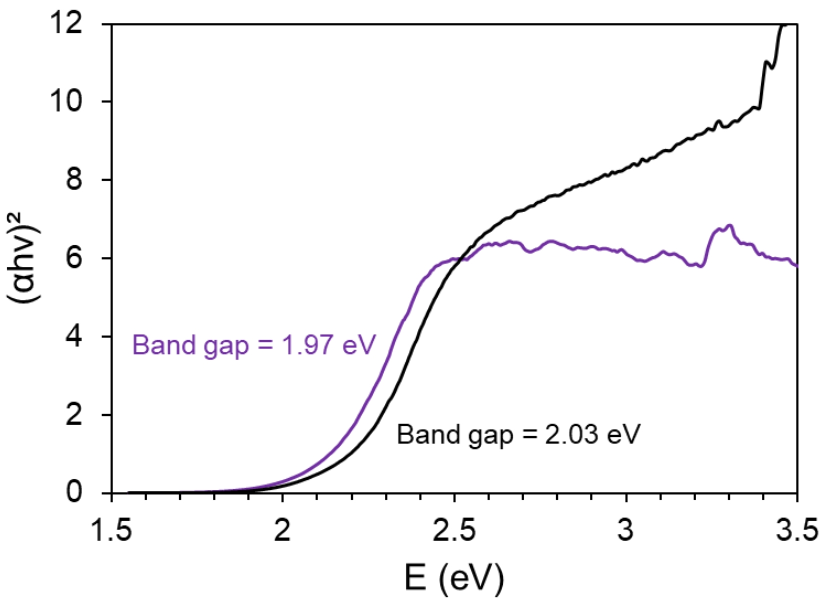

Figure S6. Band gap of IPREM-COF (black) and *I-COF-Poly-0.5* (purple).

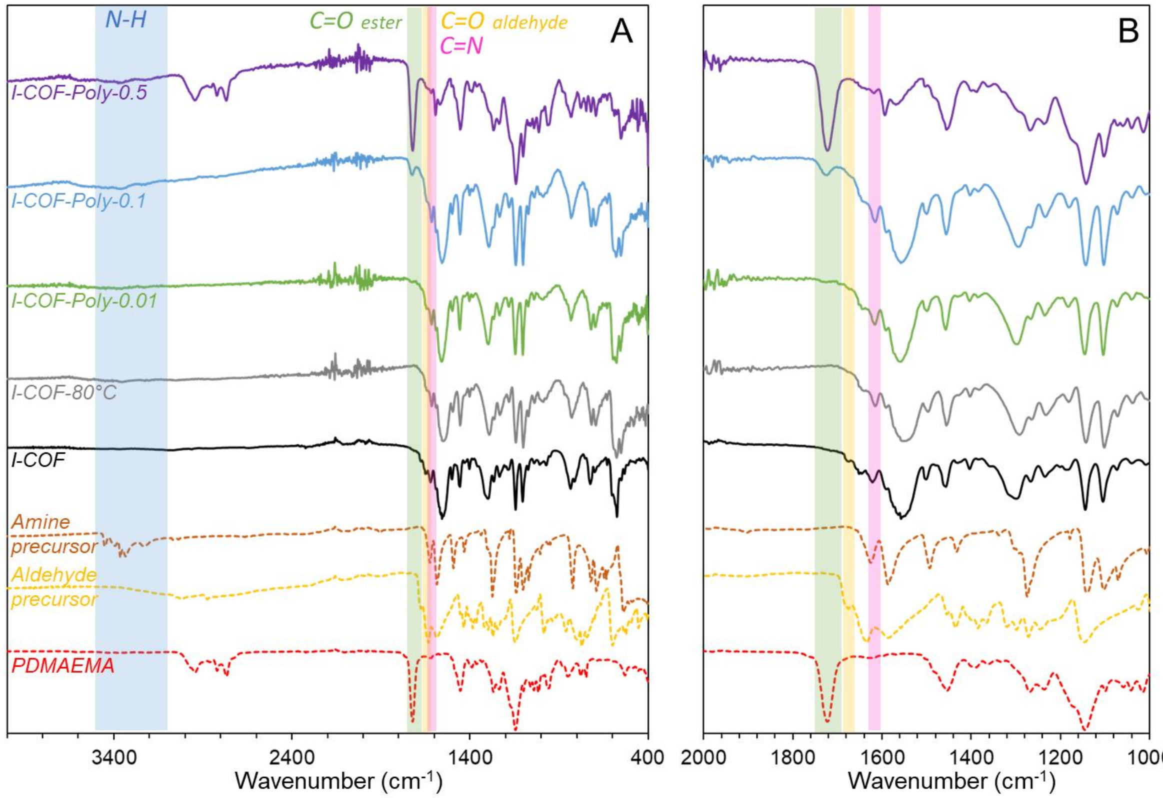

Figure S7. Infrared spectra of COFs and their precursors over the full analytical range (A) and zoomed in from 1000 to 2000 cm^{-1} (B).

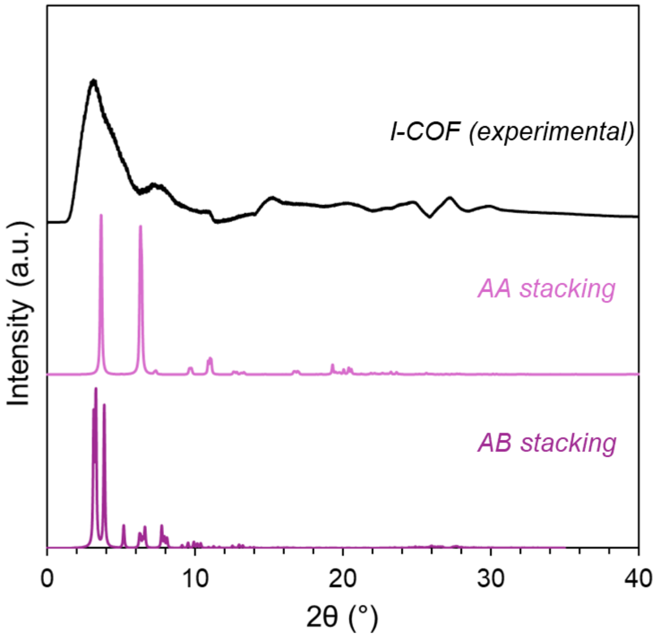

Figure S8. Comparison between experimental PXRD (black) and simulated PXRD of AA (pink) and AB (purple) stacking of *I-COF*.

Table S2. Interplanar distances *d* and *2θ* values associated with the peaks identified on the diffractograms in Fig. 2.

	**Interplanar distance *d* (Å)**				
Plane	*Pawley refinement*	*I-COF*	*I-COF-80°C*	*I-COF-Poly-0.01*	*I-COF-Poly-0.1*
(100)	27.75	28.19	22.69	23.98	23.47
(210)	11.54	11.98	12.98	12.91	12.76
(300) (330) (030)	6.48	/	7.11	6.85	6.94
(001)	4.50	/	4.59	4.64	4.59
	**2θ (°)**				
Plane	*Pawley refinement*	*I-COF*	*I-COF-80°C*	*I-COF-Poly-0.01*	*I-COF-Poly-0.1*
(100)	3.18	3.13	3.89	3.68	3.76
(210)	7.65	7.37	6.80	6.84	6.92
(300) (330) (030)	13.64	/	12.44	12.91	12.74
(001)	19.70	/	19.31	19.10	19.31

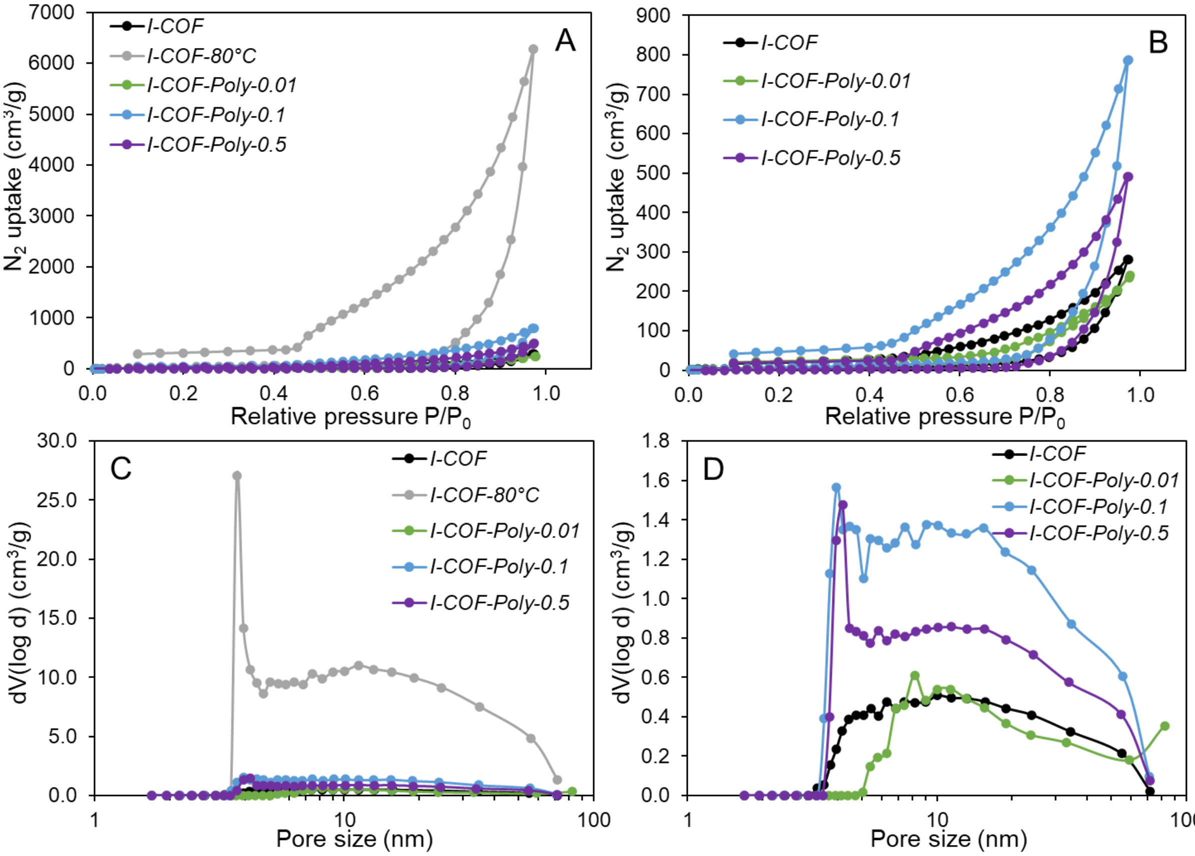

Figure S9. (A) N_2 sorption isotherms and (B) zoom in the 0-900 cm^3/g range. (C) Pore size distribution calculated from the adsorption branch of isotherms by the BJH model and (D) zoom in the 0 to 30 cm^3/g range.

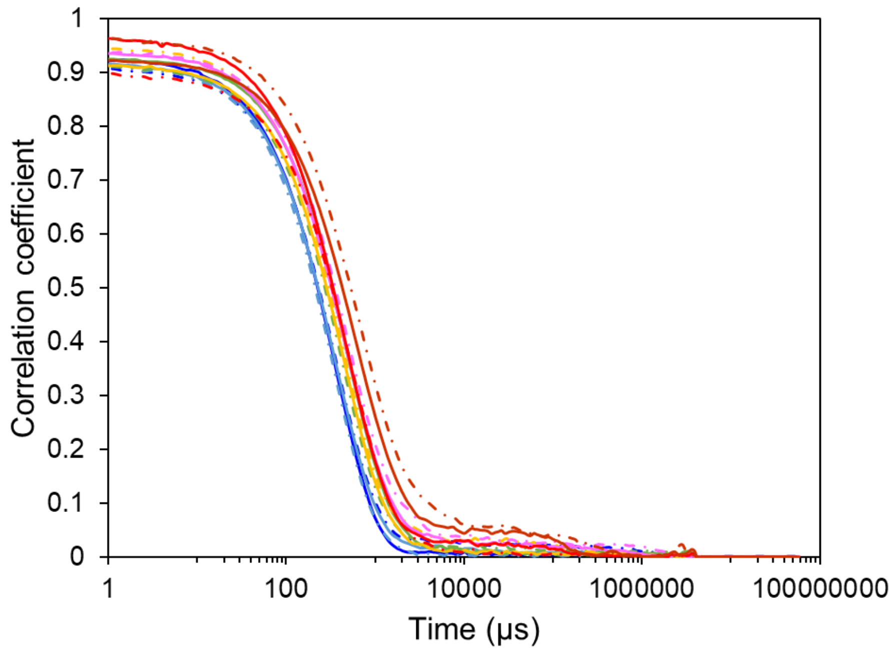

Figure S10. Correlograms of the solutions tested in photocatalysis as a function of photocatalyst concentration: 0.05 g.L^{-1} (dark blue), 0.075 g.L^{-1} (light blue), 0.10 g.L^{-1} (light green), 0.20 g.L^{-1} (yellow), 0.35 g.L^{-1} (pink), 0.50 g.L^{-1} (red), and 1.0 g.L^{-1} (brown). Solid lines correspond to the solution before photocatalysis, and dash-dotted lines after photocatalysis.

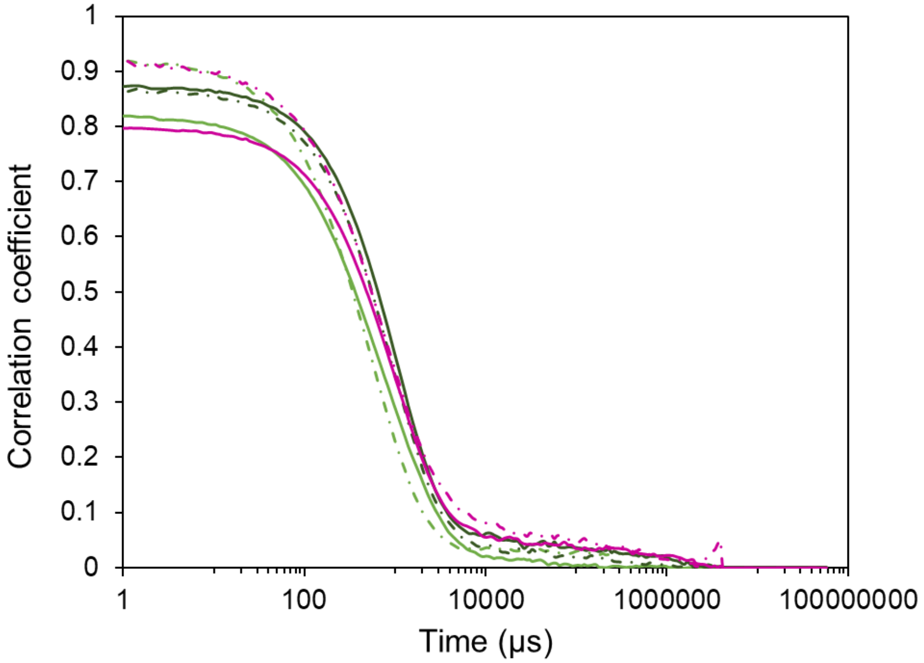

Figure S11. Correlograms of the solutions tested in photocatalysis as a function of the SED: sodium ascorbate (light green), ascorbic acid (dark green), and TEOA (purple). Solid lines correspond to the solution before photocatalysis, and dash-dotted lines after photocatalysis.

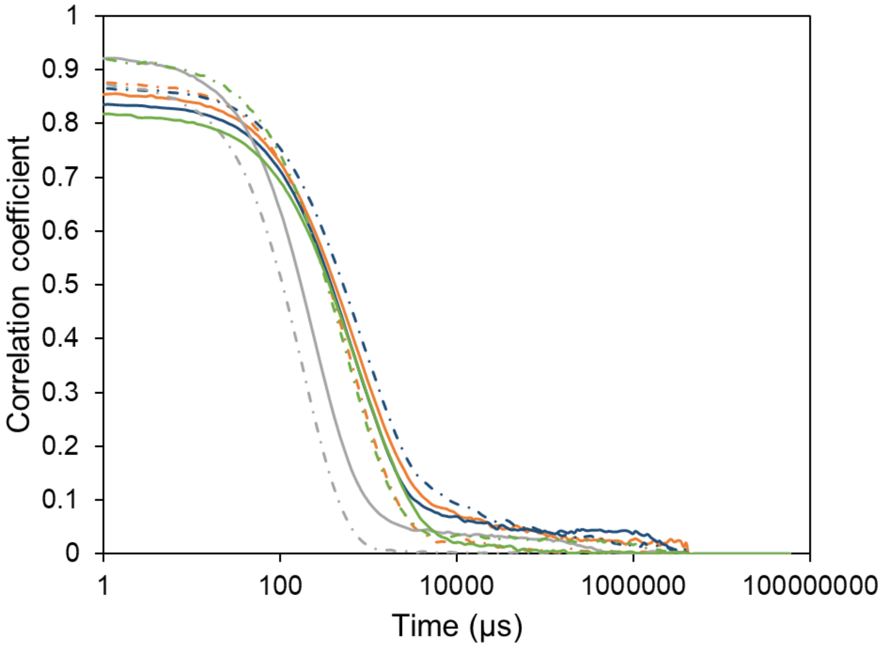

Figure S12. Correlograms of the solutions tested in photocatalysis as a function of the different co-catalysts used: H_2PtCl_6 (light green), K_2PtCl_6 (orange), $AgNO_3$ (grey), without co-catalyst (dark blue). Solid lines correspond to the solution before photocatalysis, while dash-dotted lines correspond to after photocatalysis.

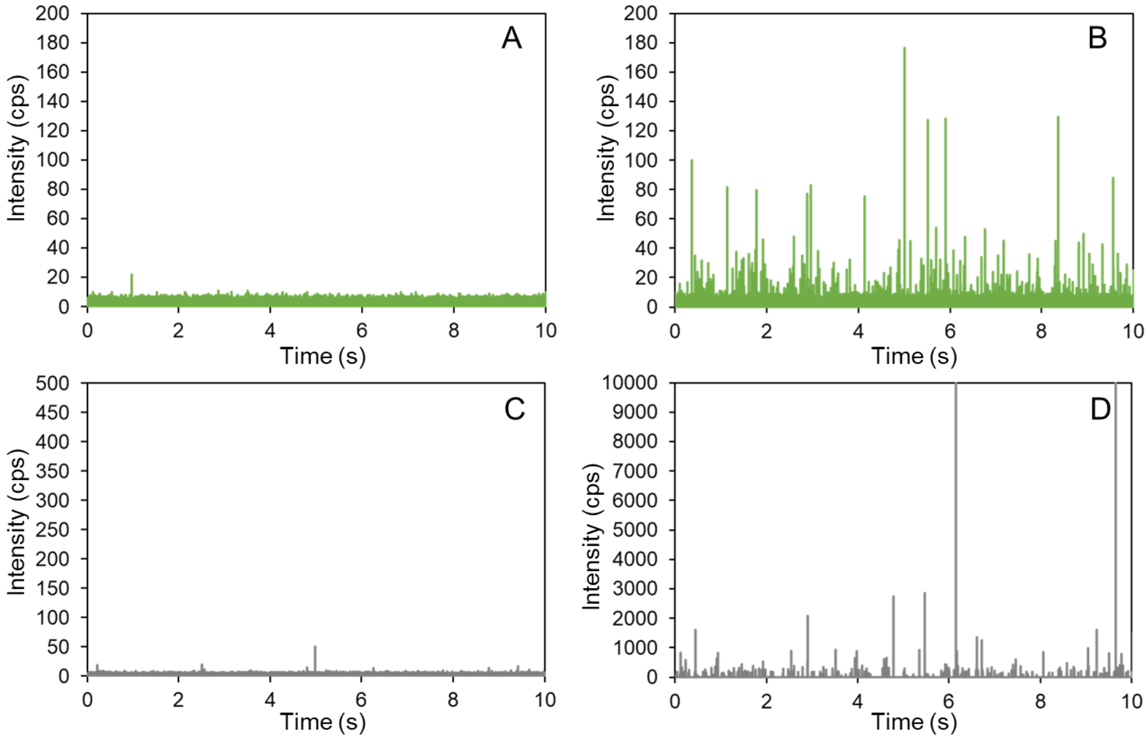

Figure S13. Raw SP-ICP-MS signals as a function of time for solutions tested under photocatalysis: in the presence of H_2PtCl_6 co-catalyst (A) before and (B) after photocatalysis; and in the presence of $AgNO_3$ (C) before and (D) after photocatalysis.

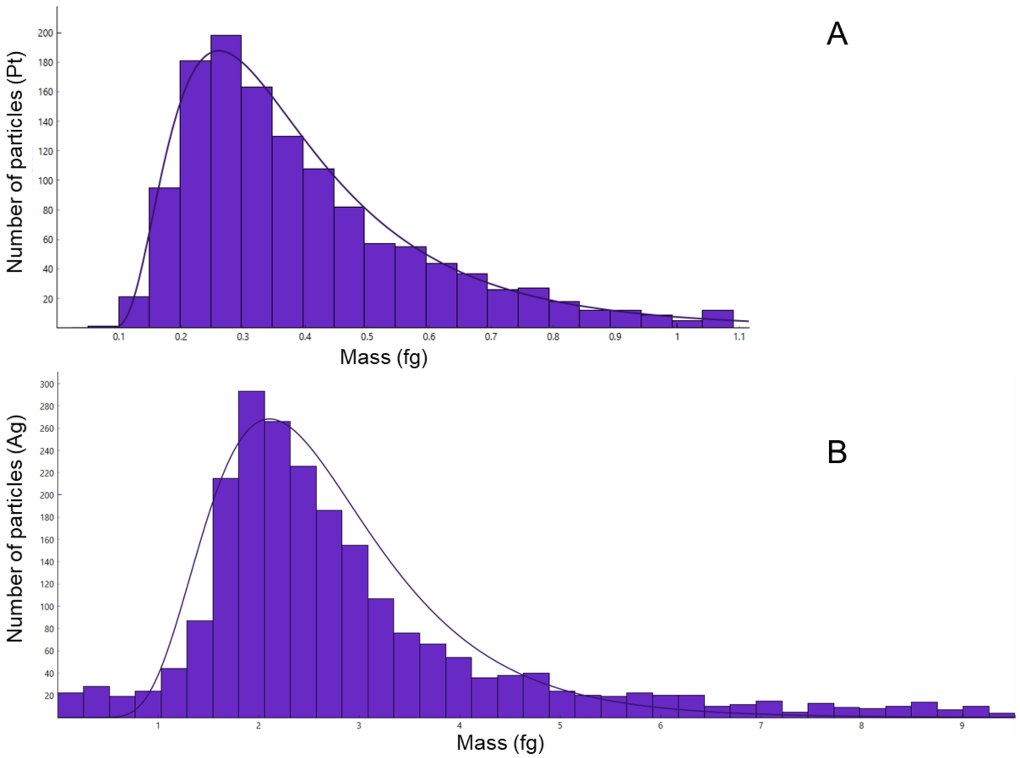

Figure S14. Average mass of (A) platinum and (B) silver detected per particles by SP-ICP-MS.
